# Green Extraction and Liposomal Encapsulation of *Inonotus obliquus* (Chaga) Extracts: Comparative Phytochemical and Antioxidant Analysis

**DOI:** 10.3390/molecules31010146

**Published:** 2026-01-01

**Authors:** Nevena Preradović, Đura Nakarada, Uroš Gašić, Jasna Simonović Radosavljević, Miloš Mojović

**Affiliations:** 1 Institute for Multidisciplinary Research, National Institute of the Republic of Serbia, University of Belgrade, 11030 Belgrade, Serbia; nevena@imsi.bg.ac.rs; 2 Center for Physical Chemistry of Biological Systems, BioScope Labs, Faculty of Physical Chemistry, University of Belgrade, 11158 Belgrade, Serbia; djura@ffh.bg.ac.rs; 3 Department of Plant Physiology, Institute for Biological Research “Siniša Stanković”, National Institute of the Republic of Serbia, University of Belgrade, 11108 Belgrade, Serbia; uros.gasic@ibiss.bg.ac.rs

**Keywords:** Chaga, green extraction techniques, EPR spectroscopy, antioxidant activity, liposomes

## Abstract

*Inonotus obliquus* (Chaga) is a medicinal basidiomycete fungus with diverse bioactive compounds and pharmacological properties. This study systematically compared green extraction techniques: maceration, ultrasound-assisted extraction (UAE), and combined supercritical CO_2_-pressurized liquid extraction (ScCO_2_-PLE), using solvents of varying polarity (water, 50%, and 70% ethanol). Chaga extracts were analyzed for phytochemical composition (HPLC-Orbitrap Exploris 120) and antioxidant activity toward DPPH and hydroxyl (^•^OH) radicals using EPR spectroscopy. The results revealed that both solvent polarity and extraction technique significantly influenced extraction efficiency and antioxidant potential. The UAE extraction method achieved the highest overall recovery of phenolic and triterpenoid compounds, with extracts obtained using 50% and 70% ethanol exhibiting the most pronounced and well-balanced radical scavenging activity (>98% toward DPPH, >91% toward ^•^OH). Correlation and PCA analyses identified phenolic and triterpenoid compounds, including fungal-specific polyphenols such as hispidin and hispolon, as key contributors to antioxidant activity. Among the extracts obtained using different solvents, the extracts with the highest overall antioxidant potential were encapsulated into liposomes and evaluated for their DPPH and ^•^OH radical scavenging. Encapsulation effectively preserved the antioxidant activity of ethanol-derived extracts, demonstrating that Chaga liposomes can maintain bioactivity while offering the advantages of controlled delivery. Combining optimized extraction with liposomal encapsulation thus represents a promising strategy to enhance the stability and practical applicability of Chaga antioxidants in nutraceutical or therapeutic contexts.

## 1. Introduction

**Figure 1:**
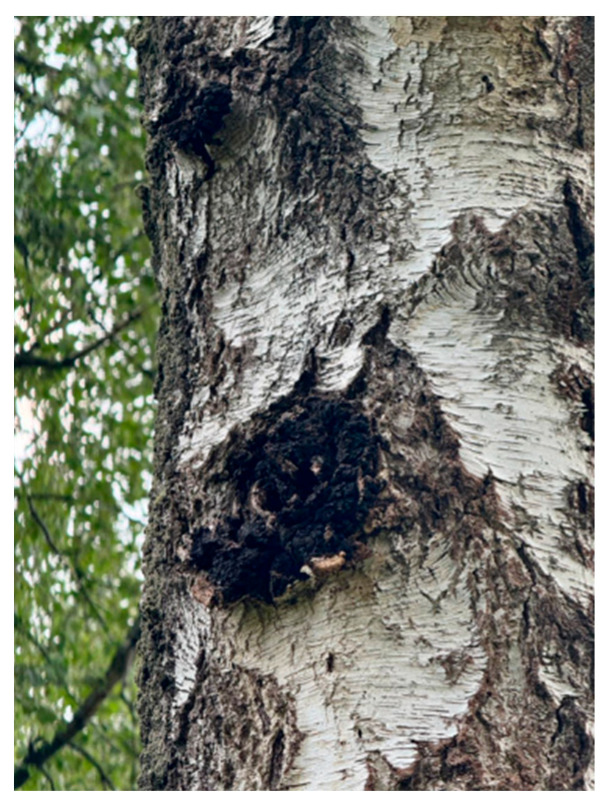
Sterile *Inonotus obliquus* (Chaga) conk on birch (*Betula* spp.) in a natural forest stand, Bryansk Oblast, Russia. Photograph taken by the author in June 2025.

*Inonotus obliquus* (Fr.) Pilát, commonly known as Chaga (Figure 1), is a parasitic basidiomycete fungus belonging to the family *Hymenochaetaceae*. It primarily colonizes mature birch trees (*Betula* spp.) throughout the northern regions of Russia, Northern Europe, North America, and Asia [1,2,3,4,5,6,7,8,9]. The fungus infects its host through wounds or bark fissures, leading to internal colonization and the eventual formation of rough, black, sterile conks (sclerotia) on the trunk. These dark, irregular structures, often misidentified as fruiting bodies, are commonly referred to as Chaga [6,7].

Chaga has been used in Siberian and Russian traditional medicine for centuries, mainly for the treatment of various health conditions [1,2,3,4,5,6,7,8,9]. It has since gained global recognition for its abundant and diverse phytochemical composition, comprising bioactive compounds such as phenolic compounds, triterpenoids (particularly betulin, betulinic acid, and inotodiol), polysaccharides (β-glucans), and the pigment melanin [1,2,3,4,5,7,8,9]. This complex chemical profile underlies its diverse biological and therapeutic effects, including antioxidant, anticancer, anti-inflammatory, immunomodulatory, antiviral, antidiabetic, hepatoprotective, and DNA-protective activities [1,2,3,4,9,10,11].

Recent studies have shown significant geographical variations in the bioactivity of Chaga. Samples originating from Russia exhibited the strongest antiproliferative and cytotoxic effects across various cancer cell lines, as well as superior antioxidant capacity, compared to samples from Finland and Thailand [12,13]. Owing to its high bioactivity and historical medicinal use, Russian Chaga was selected as the representative material for this study.

Aqueous and hydroethanolic Chaga extracts have been studied primarily because of their rich bioactive composition and their cytotoxic and antiproliferative effects in various human carcinoma cell lines [3,4,5,9,10,11,14,15,16]. To meet the increasing demand for environmentally sustainable processing, green extraction techniques such as ultrasound-assisted extraction (UAE) [17,18] and supercritical CO_2_ (ScCO_2_) extraction [19,20,21] offer efficient alternatives to conventional solvent-based methods. These approaches minimize the use of harmful organic solvents, while the choice of extraction technique significantly determines both the yield and the bioactive composition of the resulting extracts. In particular, heating and ultrasonic treatment during extraction enhance the yield of phenolic compounds and flavonoids in both aqueous and ethanolic systems [22].

Although sustainable extraction techniques have gained increasing attention in recent years, no studies have explored a combined supercritical CO_2_ and pressurized liquid extraction (ScCO_2_-PLE) approach for Chaga. Furthermore, no study has systematically compared this technique with other green extraction methods, such as maceration and UAE, using solvents of different polarity, in terms of both phytochemical composition and antioxidant activity. Therefore, this research applied and directly compared these extraction techniques for the first time, providing novel insights into their relative performance.

While Chaga’s antioxidant properties are well-documented [1,2,3,4,5,7,8,9,23,24,25,26,27], their evaluation using the advanced technique of Electron Paramagnetic Resonance (EPR) spectroscopy to measure free radical scavenging activity has not yet been reported. Traditional antioxidant assays applied to Chaga extracts, such as UV-Vis DPPH, rely on indirect spectrophotometric measurements that can be affected by sample color, turbidity, and matrix interferences. Chaga is a dark, pigment-rich material, which may compromise the accuracy of such methods. In contrast, EPR directly detects unpaired electrons in radical species, enabling specific and interference-free quantification of radical scavenging activity. EPR is a more sensitive method, capable of detecting subtle differences in radical concentrations that conventional UV-Vis assays may miss [28]. This study introduces the application of EPR spectroscopy for the comprehensive quantitative evaluation of free radical scavenging activity of Chaga extracts.

To the best of our knowledge, no studies have previously investigated the incorporation of Chaga extracts into liposomal delivery systems or examined the antioxidant activity of such formulations using EPR spectroscopy. This study therefore reports the first development and characterization of liposomal Chaga formulations and assesses their antioxidant potential in comparison with non-encapsulated extracts.

A schematic overview of the experimental workflow is presented in the Supplementary Materials in Figure S1.

## 2. Results and Discussion

### 2.1. Chemical Profiling of Chaga Extracts

The qualitative HPLC-Orbitrap Exploris 120 analysis on nine different Chaga extracts (Figure S2, Table 1) revealed a diverse profile of bioactive compounds. A total of 46 metabolites were identified. The peak areas, obtained from full scan MS spectra, of all identified compounds in all tested samples are summarized in Table S1. Data on the retention times (*t*_R_, min), molecular formulas, calculated and exact masses ([M–H]^−^, *m/z*), mass accuracy errors (Δ ppm), and major MS^2^ fragment ions of the identified compounds are summarized in Table 1.

The chemical composition of Chaga includes a wide range of structurally diverse bioactive compounds, some of which are illustrated in Figure S3. Among them, various sugars and sugar alcohols have been identified, such as mannitol and gluconic acid, which are simple carbohydrate derivatives. In addition, compounds like 2-hydroxy-1-(hydroxymethyl) ethyl 4-hydroxy-3,5-dimethoxybenzoate and Aquilarin A are glycosylated or contain sugar-like moieties.

Among the extractable compounds, polyhydroxy and polycarboxylic acids represent a significant chemical group, both aliphatic and aromatic. These include isocitric acid, dihydroxybutanedioic acid, azelaic acid, furandicarboxylic acid, and several benzenecarboxylic acid derivatives such as 2,5-dihydroxyterephthalic acid and benzenetricarboxylic acid also reported in [29,30]. Larger and more complex molecules in this category include betulinic acid, maslinic acid, and betulin-3-caffeate, reported also in [29], which combine triterpenoid structures with acidic or ester functionalities.

Several phenolic acids and their derivatives are present as well, characterized by the presence of both hydroxyl and carboxyl groups on aromatic rings. These include caffeic acid, syringic acid, 3,4-dihydroxybenzoic acid, also reported in [30], and more complex molecules such as 4-(2,6-dihydroxybenzoyl)-3-formyl-5-hydroxybenzoic acid and dimethoxyhydroquinone syringoyl-hexoside.

The extracts also contain a variety of non-acidic phenolic compounds, such as arbutin, hispidin, hispolon, gingerol, inotilone, and spinochrome A. These molecules, although lacking carboxyl groups, are rich in hydroxylated aromatic structures and contribute to the antioxidant properties of the species.

Coumarins, such as umbelliferone and scopoletin, have also been identified. These benzopyranone derivatives are known for their diverse biological activities, including anti-inflammatory and antimicrobial effects [31,32,33]. Notably, 3-hydroxy-2-oxo-2H-1-benzopyran-4,6-dicarboxylic acid represents a coumarin-related compound with additional carboxyl functionalities.

A group of structurally complex polyphenolic compounds includes molecules like gomphilactone, phelligridin J, phelligridin D, and polyporusterone B. These are often considered within the broader class of polyphenols due to their multiple hydroxylated aromatic rings.

Several terpenoids and triterpenes have been identified, including betulinic acid, maslinic acid, inonoterpene A, and polyporusterone B. These compounds are derived from isoprenoid biosynthesis and are known for their anti-inflammatory, antiviral, and anticancer properties [34,35,36,37]. Some occur as esterified forms, such as betulin-3-caffeate, which combines a triterpenoid backbone with a phenolic acid ester.

Compounds containing ester functional groups are present in the chemical profile, including benzenetetracarboxylic acid dimethyl ester, betulin-3-caffeate, also reported in [29], and 2-hydroxy-1-(hydroxymethyl)ethyl 4-hydroxy-3,5-dimethoxybenzoate. These esters often result from the combination of polyfunctional alcohols and aromatic acids, contributing to the overall structural diversity of the fungal metabolome.

The chemical profiles of Chaga extracts obtained using different extraction solvents and techniques were characterized using HPLC–Orbitrap Exploris 120 analysis. The obtained LC–MS data were used to generate a comparative chemical fingerprint of each extract, allowing for assessment of extraction selectivity toward different groups of metabolites.

Due to the absence of authentic analytical standards and correction for compound-specific ionization efficiencies, the LC–MS results provide semi-quantitative, signal-based information only. Accordingly, all reported values represent relative LC–MS signal contributions rather than absolute concentrations or true chemical composition.

**Figure 2:**
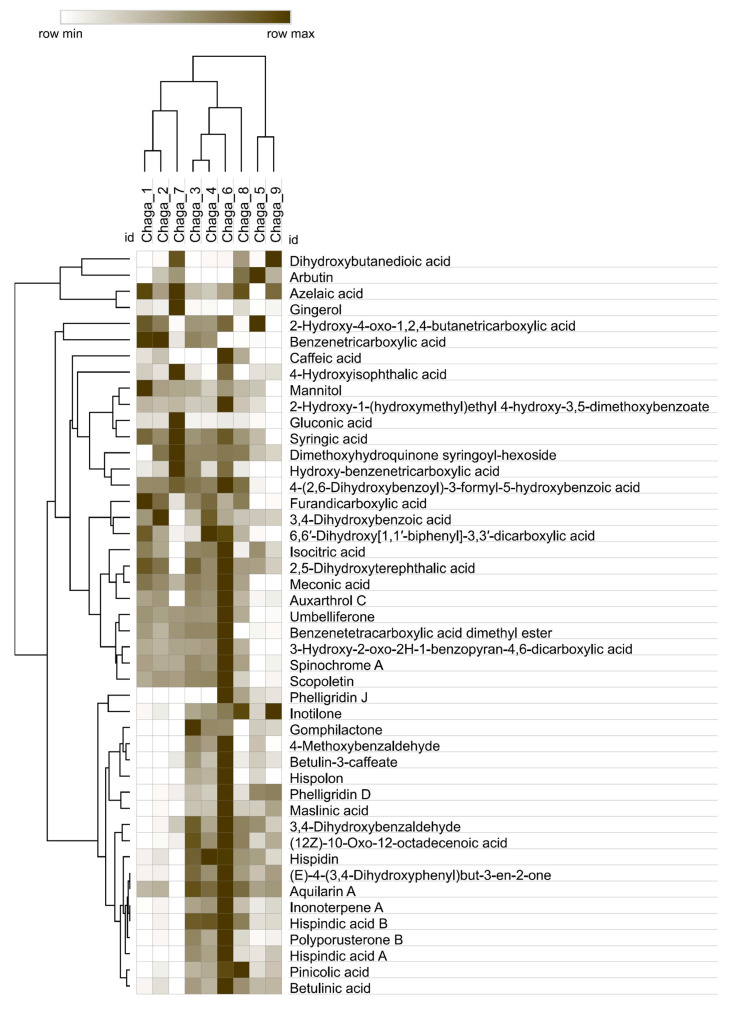
Heatmap of LC–MS data of untargeted metabolite analysis showing the relative distribution of LC–MS signal contributions in Chaga extracts obtained using different extraction methods. Samples (columns) and metabolites (rows) are arranged according to the HCA (Spearman method of cluster agglomeration). Intensities of brown color indicate the signal contribution of the compounds in samples, as indicated in the color scale. Chaga samples are defined as follows: Chaga 1—maceration, water; Chaga 2—UAE, water; Chaga 3—maceration, 50% ethanol; Chaga 4—UAE, 50% ethanol; Chaga 5—maceration, 70% ethanol; Chaga 6—UAE, 70% ethanol; Chaga 7—ScCO_2_-PLE, water; Chaga 8—ScCO_2_-PLE, 50% ethanol; Chaga 9—ScCO_2_-PLE, 70% ethanol.

The relative metabolite profiles of nine Chaga extracts obtained using different extraction techniques and solvent systems are summarized in Table S1 and grouped using hierarchical cluster analysis (HCA) for their comparative visualization (Figure 2). The heat map is based on row-wise normalized MS peak areas, and reflects relative changes in the MS response of individual metabolites across extraction conditions rather than absolute concentrations or total metabolite content. The heatmap enables detailed assessment of compound- and extraction-dependent distribution patterns of individual metabolites and overall similarities among extracts.

At the level of global sample clustering, extracts obtained using similar solvent systems tend to group together. Water-based extracts obtained by maceration (Chaga 1), ultrasound-assisted extraction (Chaga 2), and ScCO_2_-PLE (Chaga 7) show comparable overall profiles and cluster separately from ethanol extracts. Ethanol extracts (Chaga 3–6 and Chaga 8–9) form a distinct cluster, with further separation between 50% ethanol extracts (Chaga 3, Chaga 4, and Chaga 8) and 70% ethanol extracts (Chaga 5, Chaga 6, and Chaga 9), suggesting that ethanol concentration contributes to differences in relative metabolite profiles.

Highly polar primary metabolites (such as sugar alcohols) and low-molecular-weight organic acids, cluster together due to similar relative distribution patterns across the extraction conditions. Mannitol exhibits its highest relative signal contributions in the water-based maceration extract (Chaga 1), while comparatively high relative contributions are also observed in ethanol extracts, most notably in the UAE 70% ethanol extract (Chaga 6). Gluconic acid exhibits a compound-specific behavior, with a pronounced relative maximum in the ScCO_2_-PLE water extract (Chaga 7) while showing lower relative signal contributions in other water-based and ethanol extracts. Dihydroxybutanedioic acid shows higher relative signals in ScCO_2_-PLE extracts (Chaga 7–9), while lower relative contributions are observed in other ethanol and water-based extracts. Isocitric acid shows substantial relative signals across multiple extraction conditions. Its highest relative signal is observed in the UAE 70% ethanol extract (Chaga 6), with slightly lower but comparable relative contributions in Chaga 1–5 and distinctly reduced signals in ScCO_2_-PLE extracts (Chaga 7–9).

Phenolic acids and aromatic polycarboxylic acids show pronounced compound-specific variability rather than uniform solvent-driven trends. For example, syringic acid exhibits high relative signal intensities in several water-based extracts, including Chaga 1 and Chaga 7, as well as in selected ethanol extracts such as Chaga 6, indicating that its extraction is not strictly governed by solvent polarity. Caffeic acid exhibits a selective extraction pattern, showing detectable relative contributions in water-based extracts obtained by maceration and UAE (Chaga 1 and Chaga 2), with its highest relative signal observed in the UAE 70% ethanol extract (Chaga 6) and a pronounced contribution also detected in the ScCO_2_-PLE 50% ethanol extract (Chaga 8) while remaining low or negligible in the other extracts. These observations highlight that extraction efficiency for phenolic acids is strongly compound-dependent and cannot be generalized solely on the basis of solvent composition.

Coumarins and structurally related phenolic derivatives show heterogeneous distribution patterns across the extracts. Umbelliferone exhibits high relative signal intensities in several ethanol extracts (Chaga 3, Chaga 4 and Chaga 6) while showing markedly reduced relative contributions in 70% ethanol maceration and ScCO_2_-PLE extracts (Chaga 5 and Chaga 9). Scopoletin follows a similar trend, with enhanced relative signals in Chaga 3, Chaga 4 and Chaga 6, but consistently low relative signal intensities in Chaga 5 and Chaga 9. These patterns suggest that both solvent composition and extraction technique influence the recovery of coumarin-type compounds.

Hispidin-type polyphenols and phelligridin derivatives, including hispidin, hispolon, phelligridin D, and phelligridin J, form a distinct cluster characterized by very low or negligible relative signals in water-based extracts (Chaga 1, Chaga 2, and Chaga 7). Their highest relative contributions are consistently observed in ethanol extracts, particularly in the UAE 70% ethanol extract (Chaga 6), with moderate relative signals in 50% ethanol extracts (Chaga 3, Chaga 4).

Terpenoids and other lipophilic constituents, including betulinic acid, betulin-3-caffeate, maslinic acid, polyporusterone B, and pinicolic acid, exhibit the strongest dependence on solvent composition. These compounds show minimal relative contributions in aqueous extracts (Chaga 1, Chaga 2, and Chaga 7) and progressively higher relative signals with increasing ethanol concentration. The most pronounced signal contribution is observed in ethanol extract obtained by UAE (Chaga 6 and Chaga 4) and ScCO_2_-PLE (Chaga 8), confirming that low-polarity extraction environments are required for efficient recovery of triterpenoid constituents from Chaga [38,39].

Within identical solvent systems, extraction methodology further modulates the relative metabolite profiles of Chaga extracts. UAE is associated with increased relative signal intensities of several phenolic acids, coumarins, terpenoids, and hispidin-type polyphenols compared to maceration, particularly in ethanol-containing extracts [18,40]. ScCO_2_-PLE generates distinct relative profiles, characterized by selective enrichment of low-polarity constituents when ethanol is used as a modifier while showing reduced relative representation of highly polar metabolites [20].

The class-based interpretation of the heatmap indicates that solvent polarity is closely associated with the overall clustering of Chaga extracts, while extraction technique and solvent composition contribute to variations in the relative signal contribution of individual metabolites.

### 2.2. Antioxidant Activity of Chaga Extracts

The antioxidant activity (*AA*) of nine Chaga extracts was evaluated based on their radical scavenging ability against 2,2-diphenyl-1-picrylhydrazyl (DPPH) and hydroxyl (^•^OH) radicals using electron paramagnetic resonance (EPR) spectroscopy. In contrast to indirect spectrophotometric assays, EPR enables the direct detection of unpaired electrons in radical species, allowing for highly specific and interference-free quantification of radical scavenging activity, even in complex extract matrices.

After recording the EPR spectra, a characteristic single-line spectrum of the DPPH radical was observed in aqueous solution, while a well-resolved five-line spectrum was detected in ethanol. This difference arises from solvent-dependent molecular mobility and spin-spin interactions of the DPPH radical. In aqueous media, limited solubility and stronger intermolecular interactions lead to spectral broadening and effective collapse of hyperfine structure into a single line.

**Figure 3:**
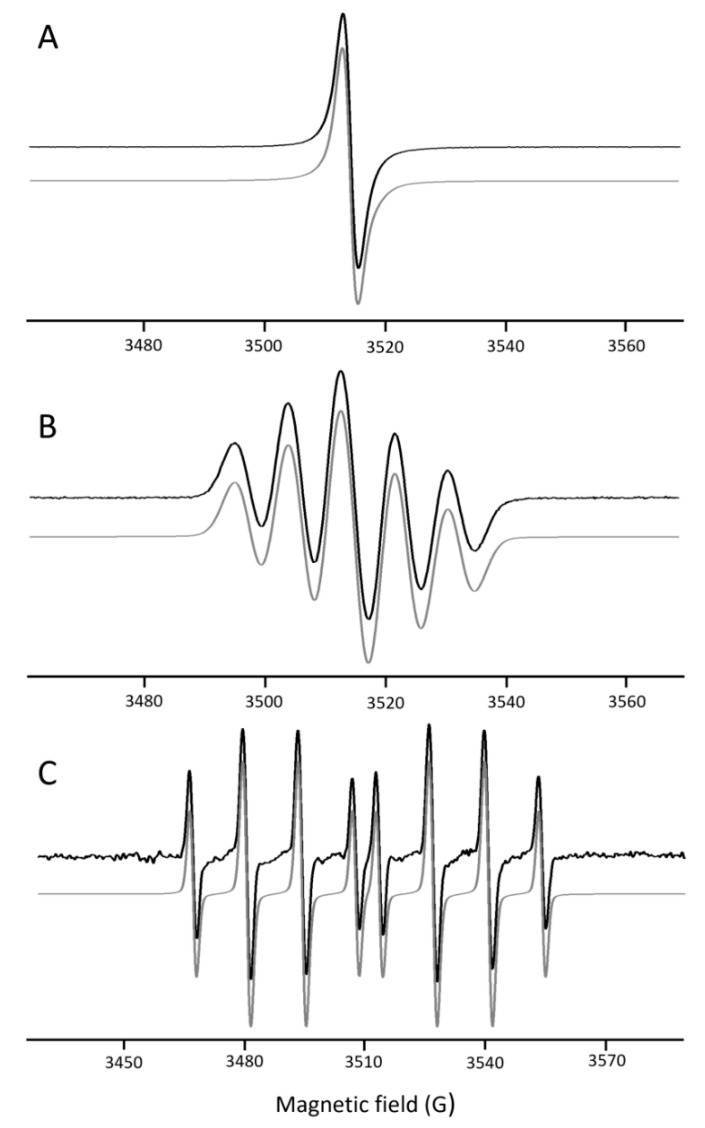
The representative Electron Paramagnetic Resonance (EPR) spectra (black line) and simulation spectra (grey line) for: (**A**) Water solution of 2,2-diphenyl-1-picrylhydrazyl (DPPH); (**B**) Ethanol solution of 2,2-diphenyl-1-picrylhydrazyl (DPPH); (**C**) 5-(Diethoxyphosphoryl)-5-methyl-1-pyrroline N-oxide hydroxyl radical adduct (DEPMPO-OH). EPR simulation parameters for the DPPH radical in water (single peak, g = 2.003) and ethanol (a^N^ [2N] = 8.62 G) were adapted from [43], whereas the parameters for the DEPMPO-OH spin adduct (a^P^ = 46.66 G, a^N^ = 13.78 G, a^H^[1H] = 13.32 G) were obtained from [44].

In the case of hydroxyl radicals, a characteristic eight-line EPR spectrum corresponding to the 5-(diethoxyphosphoryl)-5-methyl-1-pyrroline-*N*-oxide (DEPMPO)-OH spin adduct was observed [41,42]. The representative experimental EPR spectra and their corresponding simulations for the DPPH radical in water and ethanol, as well as for the DEPMPO–OH adduct, are shown in Figure 3. Representative EPR spectra illustrating the reduction in DPPH and DEPMPO-OH signals upon addition of extracts obtained using different extraction methods and solvents (water and ethanol), are shown in Figure S4.

The area under the EPR spectra was determined from the simulations, and the obtained data were used for the calculation of antioxidant activity. Table 2 presents the measured antioxidant activities of Chaga extracts against DPPH and ^•^OH radicals.

#### 2.2.1. Effect of Solvent Type

A clear trend was observed in relation to solvent type: ethanol-based extracts exhibited significantly higher antioxidant activity compared to water-based extracts. All ethanol extracts (Chaga 3–6 and Chaga 8) showed DPPH scavenging values above 90%, with the exception of Chaga 9 (15.7%). In contrast, water extracts (Chaga 1, 2, and 7) demonstrated lower antioxidant activity, particularly against ^•^OH radicals (e.g., Chaga 1: 31.5%).

The observed differences in antioxidant activity between ethanol and water extracts can be attributed to the polarity and extraction efficiency of the solvents. Ethanol, especially in aqueous mixtures (50% or 70%), is known to be more effective in extracting a wide range of phenolic compounds and other antioxidant molecules, many of which are moderately polar. In contrast, distilled water, due to its high polarity, may have limited capacity to solubilize less polar or non-polar bioactive compounds present in Chaga. As a result, water-based extracts exhibited lower overall antioxidant activity, particularly against the hydroxyl radical. This highlights the importance of solvent selection in optimizing the recovery of antioxidant constituents during extraction, as reported in previous studies [17,45,46].

#### 2.2.2. Effect of Extraction Method

Among the tested extraction methods, ultrasound-assisted extraction (UAE) consistently provided the highest and most balanced antioxidant activity, particularly when ethanol was used as the extraction solvent. For instance, Chaga 4 (UAE, 50% ethanol) and Chaga 6 (UAE, 70% ethanol) exhibited near-complete DPPH radical scavenging (98.4% and 98.5%, respectively), alongside excellent hydroxyl radical scavenging (92.7% and 91.7%). These values were slightly higher than those obtained through conventional maceration with the same solvents, suggesting that UAE may enhance mass transfer and the release of bioactive compounds, potentially through improved cell wall disruption.

When comparing UAE to maceration across all solvent systems, UAE consistently resulted in equal or superior antioxidant activity. In water-based extracts, Chaga 2 (UAE) showed improved activity over Chaga 1 (maceration), increasing DPPH scavenging from 56.2% to 65.3%, and ^•^OH scavenging from 31.5% to 45.3%. In 50% ethanol extracts, UAE (Chaga 4) yielded slightly lower DPPH scavenging compared to maceration (Chaga 3: 98.4% vs. 98.8%), but higher hydroxyl radical activity (92.7% vs. 90.6%). A similar trend was observed with 70% ethanol extracts: UAE (Chaga 6) reached 98.5% (DPPH) and 91.7% (^•^OH), slightly surpassing maceration (Chaga 5: 94.3% and 91.6%, respectively). These findings reinforce the UAE’s potential to enhance the extraction efficiency of antioxidant compounds across various solvent systems.

The comparison between maceration and UAE provides insight into the extent to which ultrasound accelerates the extraction process without reducing, and in some cases even enhancing, the bioactivity of the sample. In the present study, UAE produced comparable or slightly better antioxidant activity, as well as similar relative contents of phenolic and terpenoid compounds, all within 15 min, in contrast to 5 h required for conventional maceration.

The improved performance of the UAE may be attributed to enhanced disruption of cell walls and increased solvent penetration, which collectively could lead to more efficient mass transfer. Moreover, the UAE avoids excessive heating, thereby preserving thermolabile and volatile antioxidant compounds.

In contrast, supercritical CO_2_ extraction with Pressurized Liquid Extraction (ScCO_2_-PLE) demonstrated a more selective antioxidant profile. Although generally less effective against DPPH radicals, most notably in Chaga 9 (15.7%), ScCO_2_-PLE extracts exhibited notable activity toward the hydroxyl radical. For example, Chaga 7 (69.7%), Chaga 8 (89.6%), and Chaga 9 (89.5%) all showed pronounced ^•^OH scavenging, despite variability in DPPH performance. This selectivity, particularly toward the biologically relevant and highly reactive hydroxyl radical, may be of significant importance in biomedical applications focused on mitigating oxidative stress.

The antioxidant activity of ScCO_2_-PLE extracts demonstrated strong solvent polarity dependence. The 50% ethanol extract (Chaga 8) exhibited the highest DPPH and ^•^OH scavenging capacity, indicating optimal recovery of phenolic antioxidants at this solvent ratio. The 70% ethanol extract (Chaga 9) showed a pronounced decrease in DPPH scavenging activity (15.7%). This reduction is most likely related to the lower solubility of polar phenolics at higher ethanol levels, as well as the possible loss of more sensitive phenolic compounds during ScCO_2_-PLE. Because the DPPH assay primarily reflects the availability of phenolic hydrogen donors, a decrease in phenolic content is directly mirrored in the reduced scavenging capacity. The ^•^OH scavenging activity of Chaga 9 remained nearly identical to that of Chaga 8. Hydroxyl radical neutralization is less phenolic-dependent and involves a broader range of compounds, including melanin-like pigments, polysaccharides, amino acid residues, and lanostane-type triterpenoids. These components remain extractable at higher ethanol ratios, explaining the preserved ^•^OH scavenging capacity. These results indicate that solvent composition critically modulates the antioxidant profile of Chaga extracts by altering the balance between polar and less-polar bioactive components.

Additionally, ScCO_2_-PLE offers several practical advantages: the method uses water, minimal or no organic solvents, reducing the potential for solvent residues and making the extracts more suitable for pharmaceutical or nutraceutical use. By adjusting extraction parameters (pressure, temperature, and co-solvent composition), the technique can selectively enrich particular bioactive fractions, including highly hydroxyl-reactive compounds. This tunability, combined with its ability to preserve thermolabile compounds, makes ScCO_2_-PLE a valuable approach for generating targeted antioxidant extracts with potentially enhanced biological relevance.

Furthermore, this study represents the first application of ScCO_2_-PLE to Chaga, providing valuable insight into how this innovative technique compares with conventional extraction methods in terms of chemical composition and antioxidant activity.

#### 2.2.3. Selectivity Towards Hydroxyl Radical

An interesting finding was the selectivity of certain extracts toward the hydroxyl radical. That was particularly evident in Chaga 9 (ScCO_2_-PLE, 70% ethanol), which showed extremely low DPPH scavenging (15.7%) but high ^•^OH scavenging (89.5%). A similar trend occurred in Chaga 7 (ScCO_2_-PLE, water), where DPPH activity was 38.1%, and ^•^OH activity was significantly higher at 69.7%. This selectivity may be attributed to compositional differences-extracts obtained with polar solvents (water or aqueous ethanol) are typically richer in polyhydroxylated and polar phenolic compounds, which can efficiently chelate metal ions and neutralize highly reactive hydroxyl radicals, but are less effective toward relatively stable organic radicals like DPPH. Therefore, although overall antioxidant capacity is relevant, selectivity toward biologically relevant radicals such as ^•^OH may be of greater biomedical interest, particularly in the development of antioxidant therapies or protective formulations.

### 2.3. Correlation Between Chemical Composition and Antioxidant Activity

To explore the relationship between chemical fingerprints and antioxidant potential, correlation analysis and principal component analysis (PCA) were applied.

Correlation analysis at the level of individual metabolites (Table 3) revealed relationships among individual metabolites, and the measured scavenging activities toward DPPH and hydroxyl (^•^OH) radicals, taking into account statistical significance (*p* < 0.05).

For DPPH scavenging (Table 3), hispolon (r = 0.73, *p* = 0.026), 4-methoxybenzaldehyde (r = 0.72, *p* = 0.029), and hispidin (r = 0.66, *p* = 0.053) showed positive associations, although the latter is marginally above the 0.05 threshold. Strong negative correlations were observed for azelaic acid (r = −0.94, *p* = 0.00016) and dihydroxybutanedioic acid (r = −0.82, *p* = 0.0068), indicating that these metabolites may suppress DPPH radical scavenging.

For hydroxyl radical scavenging (Table 3), hispolon (r = 0.81, *p* = 0.0081), dihydroxybutanedioic acid (r = 0.81, *p* = 0.0081), and 4-methoxybenzaldehyde (r = 0.80, *p* = 0.0096) showed statistically significant positive correlations, while azelaic acid (r = −0.86, *p* = 0.0029) and polyporusterone B (r = −0.79, *p* = 0.011) were significantly negatively correlated. These results indicate that both polyphenolic compounds and certain organic acids play important roles in ^•^OH scavenging.

The antioxidant capacity of Chaga extracts is closely linked to specific metabolites rather than broad compound classes, with statistical significance confirming the key contributors. Hydroxyl radical scavenging is more strongly associated with both polyphenols and selected organic acids than DPPH scavenging in this dataset, highlighting differences in the mechanisms of radical neutralization.

PCA revealed that the primary sources of chemical variance (PC1 and PC2) were closely associated with antioxidant activity. DPPH radical scavenging showed a strong positive correlation with PC2 (r ≈ 0.68), while ^•^OH scavenging was inversely correlated with PC1 (r ≈ −0.82), indicating distinct chemical determinants for each antioxidant assay. These trends were consistent across correlation plots and PCA score visualizations (see Figures S5–S8 in Supplementary Materials).

**Figure 4:**
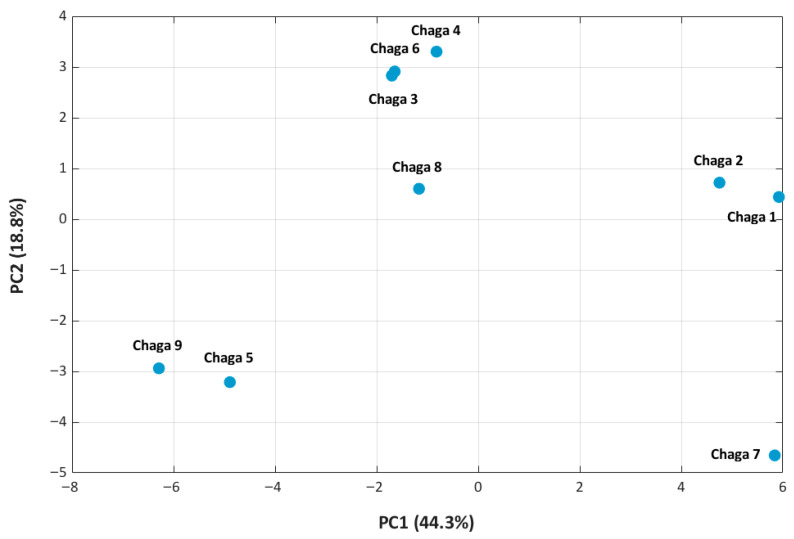
PCA score plot (PC1 vs. PC2) of nine Chaga extracts showing metabolic variation related to antioxidant activity. Chaga 3, 4, and 6 clustered with high DPPH and ^•^OH activity; Chaga 5 and 9 showed high ^•^OH/low DPPH activity; Chaga 7 was an outlier with low activity, while Chaga 1, 2, and 8 displayed distinct or intermediate profiles.

Further analysis of the score plot (PC1 vs. PC2) separated extracts enriched in identified hispidin-type phenolics and related metabolites from those with higher relative LC–MS signal contributions of sugars, sugar alcohols, and small aliphatic acids (Figure 4). Extracts with negative PC1 scores tend to have higher levels of hispidin-type phenolics, such as hispidin and hispolon. These compounds are known to be efficient at scavenging hydroxyl radicals, which fits well with the strong inverse correlation we found between PC1 and ^•^OH activity (r ≈ −0.82) [47,48,49].

PC2 captured differences mainly related to the relative LC–MS signal contributions of small phenolic acids and aromatic compounds. These metabolites act as hydrogen donors, and extracts with higher PC2 scores generally exhibited stronger DPPH scavenging. This is consistent with the positive correlation between PC2 and DPPH activity (r ≈ 0.68) [50,51].

**Figure 5:**
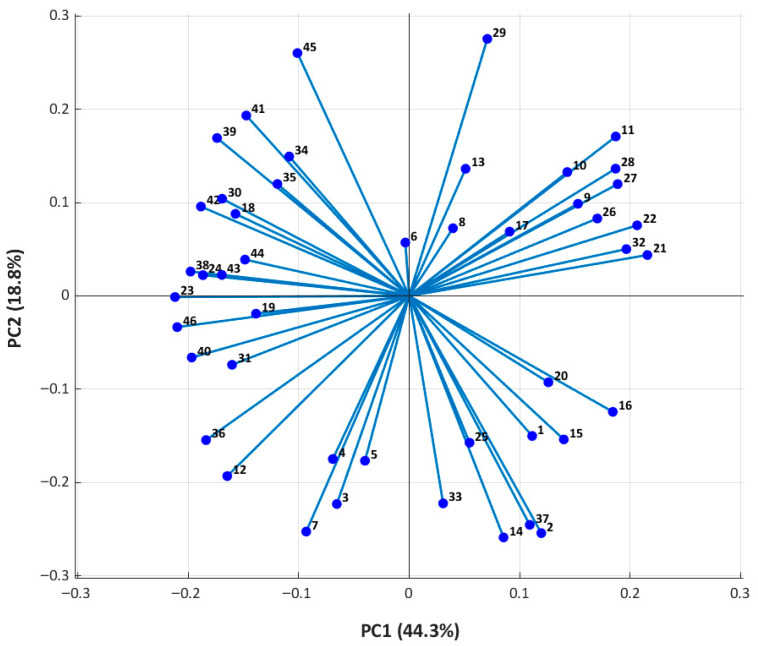
PCA biplot of bioactive compounds (PC1 vs. PC2). Vectors indicate compound contributions. The axes represent standardized coordinates in the PCA space, showing the relative positions of compounds (vectors) along the first two principal components. Values indicate the contribution of each variable to the principal components, with positive and negative values representing directions in the reduced multivariate space. Upper-left quadrant (PC1−/PC2+) contains hispidin, hispolon, hispidinic acids, and inonoterpene A, linked to strong dual antioxidant activity. Lower-right quadrant (PC1+/PC2−) contains sugars, sugar alcohols, and polyhydroxy acids, associated with weakly active extracts. The numbers refer to the compounds shown in Table 1 in the corresponding order.

The PCA biplot (Figure 5) provided a compound-level perspective, showing how individual metabolites contribute to the observed antioxidant trends.

Extracts in the upper-left quadrant, rich in hispidin-type polyphenols and some oxygenated terpenoids, showed high ^•^OH scavenging and moderate DPPH activity. Extracts in the lower-right quadrant contained more sugars and aliphatic acids and showed only weak antioxidant activity [52,53]. Other compounds, such as small aromatic acids, contributed primarily to DPPH activity. These trends also reflect how extraction conditions affect the chemical profile. Using intermediate ethanol concentrations improved the extraction of phenolic antioxidants, whereas higher ethanol levels lowered both phenolic content and antioxidant activity [54,55]. These results indicate that the antioxidant potential of Chaga extracts is closely linked to the chemical nature and redox properties of their metabolites [56,57].

### 2.4. Selection of Optimal Extracts for Liposomal Incorporation

Based on the combined assessment of antioxidant potency and compositional profile, the UAE extracts obtained with 50% and 70% ethanol (Chaga 4 and Chaga 6) were identified as the most promising candidates for further formulation into liposomal carriers. These extracts demonstrated the highest overall radical scavenging capacity against both DPPH and hydroxyl radicals, reflecting their broad-spectrum antioxidant potential.

Interestingly, although Chaga 4 did not possess the absolute highest content of phenolic or triterpenoid compounds, it exhibited the best overall antioxidant performance. This suggests that the observed bioactivity may be driven by synergistic interactions between multiple compound groups rather than the dominance of a single class. On the other hand, Chaga 6 was characterized by the highest relative triterpenoid content, which aligned well with its strong activity against both radicals. These findings highlight the importance of compound synergy and optimal extraction conditions in maximizing the functional potential of Chaga extracts.

In contrast, the ScCO-PLE extracts, particularly Chaga 7 (water-based) and Chaga 9 (70% ethanol), exhibited selective antioxidant responses, with relatively low DPPH scavenging but pronounced hydroxyl radical neutralization. Such selectivity may offer unique advantages in therapeutic contexts where hydroxyl radical suppression is particularly relevant, such as in neurodegenerative or inflammatory conditions.

Correlation analyses at both the compound group and individual molecule level further supported the extract selection. Phenolic acids, sugars, and sugar alcohols showed weak or even negative correlations with antioxidant activity, especially in assays targeting hydroxyl radicals. Conversely, compounds such as hispidin, hispolon, hispidinic acid derivatives, and betulin-3-caffeate showed strong positive correlations with radical scavenging, confirming their key roles in the observed bioactivity.

These results validate the prioritization of Chaga 4 and Chaga 6 for future studies. They combine high antioxidant capacity with a rich and synergistic phytochemical composition, extracted efficiently and rapidly via UAE. Furthermore, the radical-specific activity exhibited by selected ScCO_2_-PLE extracts emphasizes the potential of customized extraction approaches designed to match the oxidative stress profiles relevant to biomedical and nutraceutical use.

### 2.5. Liposomal Characterization and Antioxidant Activity

For each solvent, the extract exhibiting the highest antioxidant capacity (Chaga 7, Chaga 4, and Chaga 6) was selected for liposomal formulation.

**Figure 6:**
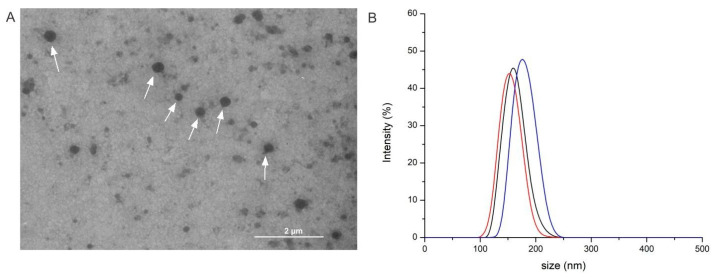
(**A**) TEM image of liposomes loaded with 70% ethanol Chaga extract (Chaga 6) recorded at magnification of 5600× (scale bar = 2 μm). Liposomes are indicated with arrows. (**B**) DLS size distribution of liposomes loaded with: (black line) ScCO_2_-PLE water Chaga extract (Chaga 7), (red line) 50% ethanol Chaga extract (Chaga 4), (blue line) 70% ethanol Chaga extract (Chaga 6). The average particle sizes were 153 nm, 160 nm, and 175 nm, respectively.

The morphology of Chaga liposomes obtained by Transmission Electron Microscopy (TEM) is presented in Figure 6A. The observed spherical structures exhibit morphology characteristic of successfully formed liposomes. The size distribution of the liposomal formulations was measured by the Dynamic Light Scattering (DLS) method (Figure 6B). Liposome sizes were observed to increase with decreasing extract polarity: 153 nm for the water extract (Chaga 7), 160 nm for the 50% ethanol extract (Chaga 4), and 175 nm for the 70% ethanol extract (Chaga 6). The zeta potential of liposomes was measured by the Electrophoretic Light Scattering (ELS) method. All liposomes showed a moderately negative zeta potential (−25.0 mV for Chaga 7, −24.1 mV for Chaga 4, and −30.7 mV for Chaga 6), indicating good colloidal stability.

These results are consistent with the structural organization of liposomes [58], where hydrophilic constituents preferentially localize within the aqueous core and allow for tighter bilayer packing, whereas less-polar or hydrophobic components, present in ethanol extracts, tend to partition into the lipid membrane region, perturbing bilayer structure and increasing overall vesicle size [59].

The liposomal formulations showed high encapsulation efficiency (*EE%*), with only a low fraction of free (non-entrapped) compounds detected, confirming effective incorporation of the active substances from the extracts (Table S2). For physical stability, the changes in particle size and zeta potential over 7 days are summarized in Table S3. After 7 days of storage at 4 °C, the mean particle size increased only marginally, confirming the maintenance of a homogenous size distribution and minimal aggregation. Similarly, the zeta potential value remained stable, indicating that the electrostatic repulsion between liposomes was maintained. These minimal changes demonstrate satisfactory colloidal stability of the liposomal formulation for short-term storage, which is essential for ensuring a consistent release profile and practical application.

To evaluate the radical scavenging capacity of liposomes encapsulating Chaga extracts, EPR spectroscopy was employed using DPPH and ^•^OH radicals, following the same procedure as described in Section 2.2. The radical scavenging abilities of these liposomal formulations are summarized in Table 4.

Among the tested samples, liposomes containing the Chaga 4 extract (UAE, 50% ethanol) demonstrated the highest overall antioxidant efficiency: 93.5 ± 0.5% for DPPH, and 82.9 ± 1.6% for ^•^OH. Chaga 6 liposomes also exhibited pronounced activity, while Chaga 7 liposomes showed lower scavenging toward DPPH and ^•^OH radicals. These results indicate that ethanol-based extraction yields extracts with high antioxidant potential, which remain well preserved after encapsulation.

### 2.6. Effect of Liposomal Encapsulation on Antioxidant Activity

By comparing Table 2 and Table 4, a noticeable difference in antioxidant activities can be observed between free and liposome-formulated Chaga extracts. When incorporated into liposomes, the antioxidant potential of the *Chaga* extracts changed notably. Liposomes containing UAE-derived extracts (Chaga 4 and Chaga 6) retained high DPPH and ^•^OH scavenging activities, with only slight reductions compared to the free extracts. In contrast, liposomes containing the ScCO_2_-PLE extract (Chaga 7) exhibited very low DPPH and ^•^OH scavenging capacities (Table 5).

The reduced antioxidant activity observed in the liposomal formulations may be attributed to the lipid bilayer barrier of the liposome, which can limit the immediate interaction between encapsulated compounds and free radicals in solution. This effect was most evident for the Chaga 7 liposomes loaded with water extract, in which active constituents are localized mostly in the core and thus less accessible/exposed. Since lecithin liposomes demonstrated negligible radical-scavenging activity [60], it can be concluded that the obtained antioxidant activity originates predominantly from the encapsulated Chaga extract.

Nevertheless, the retention of high scavenging activity of ethanol extracts (Chaga 4 and Chaga 6) demonstrates that the liposomal system effectively preserves the antioxidant potential of the encapsulated extracts.

Despite the slight reduction in DPPH and ^•^OH scavenging observed for liposomal formulations, the liposomal encapsulation effectively preserves the antioxidant potential of ethanol-derived Chaga extracts (Chaga 4 and Chaga 6). This demonstrates that the liposomal system can maintain the functional activity of the encapsulated extracts while protecting them from potential degradation. These results underscore the potential of liposomal formulations as a promising strategy to enhance the stability and applicability of Chaga antioxidants in nutraceutical or therapeutic contexts.

## 3. Materials and Methods

### 3.1. Chemicals and Materials

Hydrogen peroxide, absolute ethanol, methanol, and 2,2-diphenyl-1-picrylhydrazyl (DPPH) were purchased from Sigma Aldrich (Schnelldorf, Germany). 5-(Diethoxyphosphoryl)-5-methyl-1-pyrroline-N-oxide (DEPMPO) was purchased from Focus Biomolecules (Plymouth Meeting, PA, USA). Chloroform was purchased from Lach:Ner (Neratovice, Czech Republic). Lecithin (Emulmetic 320) was purchased from Dragonspice Naturwaren (Reutlingen, Germany). Deionized water was purchased from Lonza (Verviers, Belgium). Gas-permeable Teflon capillary tubes were obtained from Zeus Industries (Orangeburg, SC, USA). Formvar on 3 mm 200 Mesh Cu Grids was purchased from Agar Scientific (Rotherham, UK).

The dried Chaga (*Inonotus obliquus*) sclerotia were purchased from *Farmacvet* (Krasnogorsk, Russia).

### 3.2. Sample Preparation

Before extraction, dry Chaga material was milled to a uniform powder using an electric mill (Siemens, Munich, Germany) for 5–7 min. The particle size distribution was verified using a 250–500 µm sieve to ensure sample homogeneity. This particle size range was chosen to provide a consistent and reproducible surface area, facilitating efficient mass transfer and reducing variability among extraction experiments.

### 3.3. Extraction Procedure

Chaga was subjected to three extraction techniques: maceration, ultrasound-assisted extraction (UAE), and supercritical CO_2_ with pressurized liquid extraction (ScCO_2_-PLE). In all three methods, water, 50% ethanol, and 70% ethanol were used as solvents or co-solvents, in a solid-to-liquid ratio of 1:10 (*w/v*). Water was used to reflect the traditional preparation of Chaga, while 50% and 70% ethanol were selected as standard solvents for polyphenol extraction, providing similar polarity yet enhanced solubility for a broader range of antioxidant compounds. These techniques were selected to maximize the recovery of both polar and non-polar bioactive compounds while avoiding the use of hazardous reagents and minimizing heat exposure and sample degradation [61]. Samples were extracted and labeled according to Table 6.

#### 3.3.1. Maceration

A 3 g portion of powdered *Chaga* was transferred into a 50 mL volumetric flask, followed by the addition of the appropriate solvent (water, 50% ethanol, or 70% ethanol) in a solid-to-liquid ratio of 1:10 (*w/v*). The mixture was placed on a temperature-controlled orbital platform shaker (Heidolph Unimax 1010) and agitated at 40 °C for 5 h. After extraction, the samples were centrifuged at 9000 rpm for 10 min (Eppendorf Centrifuge 5804 R, Hamburg, Germany). The resulting supernatant was collected into a clean 50 mL volumetric flask and stored at −80 °C until further analysis. The method was adapted from [62,63].

#### 3.3.2. Ultrasound-Assisted Extraction (UAE)

A 3 g portion of powdered Chaga was transferred into a 50 mL volumetric flask, followed by the addition of the appropriate solvent (water, 50% ethanol, or 70% ethanol) in a solid-to-liquid ratio of 1:10 (*w/v*). The flask was placed into the ultrasonic chamber of a high-power sonicator (950 W) (LHDM502 Ultasonic Homogenizer, Colo Lab Experts, Polje ob Sotli, Slovenia), with the probe immersed to half the depth of the mixture. Sonication was performed at 50% amplitude, with the temperature alarm set to 40 °C, although the actual sample temperature remained between 20 and 25 °C due to short exposure. Three consecutive 5 min sonication cycles were applied, consisting of 10 s ultrasonic pulses separated by 2 s cooling intervals. After extraction, the samples were centrifuged at 9000 rpm for 10 min (Eppendorf Centrifuge 5804 R, Hamburg, Germany), and the resulting supernatant was transferred to a clean 50 mL volumetric flask and stored at −80 °C until further analysis. The method was adapted from [64].

#### 3.3.3. Combined Supercritical CO_2_ and Pressurized Liquid Extraction (ScCO_2_-PLE)

A 10 g portion of powdered Chaga was weighed and loaded into a 500 mL extraction chamber of a supercritical fluid extractor (Superex F-500, Karatay/Konya, Turkey). Subsequently, 100 mL of solvent (water, 50% ethanol, or 70% ethanol) was added, corresponding to a solvent-to-solid ratio of 10:1 (mL/g). The extraction cell was securely sealed, the pressure was set to 250 bar, and the temperature was maintained at 40 °C.

The extraction parameters were selected based on our previously optimized and validated pressurized extraction protocol using the same extraction system, which demonstrated efficient recovery of bioactive compounds while preserving thermolabile constituents [65]. The relatively low extraction temperature (40 °C) was chosen to minimize thermal degradation of temperature-sensitive phenolic and polysaccharidic compounds while maintaining adequate solvent diffusivity and mass transfer. The applied pressure (250 bar) ensured supercritical CO_2_ conditions and enhanced solvent penetration into the solid matrix, particularly in the presence of polar co-solvents, thereby improving extraction efficiency.

Upon reaching the set pressure and temperature, extraction was performed over a total period of 90 min and consisted of two phases: an initial static (soaking) phase of 30 min, followed by a dynamic extraction phase of 60 min. This extraction regime was selected based on prior experience indicating that the combination of a static phase, allowing for solvent–matrix equilibration, followed by a dynamic phase, promoting continuous solute removal, provides an effective balance between extraction yield and process efficiency, with no substantial improvement observed upon further extension of extraction time. The resulting extract was collected dropwise into a collection flask and stored at −80 °C until further analysis.

### 3.4. Liposome Preparation and Characterization

Liposomes were prepared by combining the thin-film hydration and sonication method using lecithin as the phospholipid component. All extracts were pre-filtered through a 0.22 µm membrane filter (FiltraTECH, Saint-Jean-de-Braye, France). The mixture was thoroughly homogenized, and the solvents were subsequently evaporated under reduced pressure using a rotary vacuum evaporator (IKA RV3 V, IKA, Staufen, Germany) until a dry lipid film was formed. The resulting lipid film was then hydrated with ultrapure water (18 MΩ·cm) and vortexed (IKA Vortex 3, IKA, Staufen, Germany) to detach it from the flask walls. The suspension was subjected to brief ultrasonic treatment in an ultrasonic water bath (ACP-120H, MRC Lab, Holon, Israel), followed by high-power sonication (950 W; LHDM502, Colo LabExperts Equipment, Novo Mesto, Slovenia) to reduce the liposome size and improve homogeneity.

The particle size and zeta potential of the liposomal formulations were determined by DLS and ELS using a Malvern Zetasizer Nano ZS90 (Malvern Instruments, Malvern, UK). Morphology of the obtained liposomes was examined by TEM using a Philips electron microscope CM12 (Amsterdam, The Netherlands) operated at 80 kV. Samples were prepared by air-drying of a liposome dispersion drop on Formvar-coated copper grids (3 mm, 200 mesh).

### 3.5. Encapsulation Efficiency and Stability of Liposomal Formulations

Encapsulation efficiency (*EE%*) of the Chaga extracts within liposomal formulations was determined using an indirect UV–Vis spectrophotometric method (UV spectrophotometer UV-2501PC, Shimadzu, Kyoto, Japan) based on relative absorbance measurements, adopted from the literature [66]. Since complex plant extracts contain multiple UV-absorbing constituents, absorbance was used as a proxy for the total extract content. Briefly, the UV–Vis absorption spectrum of each free extract solution used for liposome preparation was first recorded, and the integral area corresponding to the characteristic absorption spectrum (220–600 nm) of polyphenolic compounds was determined and defined as the total extract content (*A_total_*). Following liposome preparation, suspensions were centrifuged at 17,500 rpm for 45 min at 4 °C (Sorvall RC-5B superspeed refrigerated centrifuge, Thermo Fisher Scientific Inc., Waltham, MA, USA) to separate liposomes from the non-encapsulated (free) extract. The integral area of the collected supernatant was measured and defined as *A_free_*.

Encapsulation efficiency (1) was calculated according to the following equation:

(1) $$EE\left(\%\right)=\;\frac{A_{total}-\;A_{free}}{A_{total}}\;\times 100$$

The physical stability of the liposomal formulations was evaluated by monitoring changes in particle size and zeta potential over time. Liposome samples were stored under controlled conditions, and DLS and zeta potential measurements were repeated after 7 days using the same experimental settings as for freshly prepared samples. Stability was assessed by comparing the particle size distribution and zeta potential values at day 0 and day 7, with minimal changes indicating satisfactory colloidal stability of the liposomal systems.

### 3.6. LC–MS Characterization-Chemical Composition of Chaga Extracts

The qualitative and semi-quantitative chemical profile of the obtained extracts was determined using the Liquid Chromatography-Mass Spectrometry (LC–MS) method (Vanquish™ Core system (Thermo Fisher Scientific, Bremen, Germany) coupled to an Orbitrap Exploris 120 mass spectrometer (Thermo Fisher Scientific, Bremen, Germany)). The extracts were lyophilized to dryness (Innova FD10S Freeze Dryer, Qingdao, China) and subsequently re-dissolved in 80% methanol at a concentration of 10 mg/mL. The resulting solutions were filtered through 0.22 µm syringe filters prior to LC–MS analysis. Separation was carried out on an analytical Syncronis C18 column (100 × 2.1 mm, particle size 1.7 µm). Chromatographic and mass spectrometric parameters were applied as previously described in the literature [67]. The tentative identification of phenolic compounds was based on monoisotopic mass and MS^2^ fragmentation patterns, with confirmation via comparison to previously reported fragmentation data [68,69,70,71]. ChemDraw software (v12.0, CambridgeSoft, Cambridge, MA, USA) was used to calculate the theoretically accurate masses, while Xcalibur software (v2.1, Thermo Fisher Scientific, Waltham, MA, USA) was used for instrument control, data acquisition, and qualitative analysis.

### 3.7. Determination of Antioxidant Activity Towards DPPH Radicals

The radical scavenging efficiency of the Chaga extracts and liposomes was assessed using the DPPH assay and EPR spectroscopy [72]. Briefly, 1 μL of the extract (or liposomes) was added to 28 μL of the corresponding solvent (water or ethanol), followed by the addition of 1 μL of 3.17 mM DPPH solution prepared in ethanol. After a 2 min incubation, the EPR signal was recorded. A control measurement was performed by adding 1 μL of the solvent instead of the extract.

EPR spectra were recorded using a Bruker ELEXSYS-II E540 spectrometer (Bruker, Rheinstetten, Germany) operating in the X-band range, with the following parameters: magnetic field center at 3500 G, microwave power 10 mW, microwave frequency 9.85 GHz, modulation frequency 100 kHz, and modulation amplitude 1 G.

The antioxidant activity of the extract was calculated using Equation (2):

(2) $$AA(\%)=\frac{Ic-Ie}{Ic}\times 100$$

where *Ic* and *Ie* are the double integral values of the EPR spectra for the control and the extract-treated sample, respectively. The double integral value corresponds to the total number of spins of the unpaired electrons, and the decrease in this parameter upon addition of the extract reflects its radical scavenging activity.

### 3.8. Determination of Antioxidant Activity Towards Hydroxyl Radicals

Hydroxyl radicals (^•^OH) were generated in the system via UV photolysis of hydrogen peroxide and detected using the spin-trap method, adopting a procedure similar to the one available in the literature [41,42]. The control EPR signal for hydroxyl radicals was generated by mixing 1 μL of the 105 mM spin trap DEPMPO, 27 μL of distilled water, and 2 μL of 21.5 mM hydrogen peroxide (H_2_O_2_) solution, followed by UV irradiation to induce radical formation. The prepared mixture was exposed to UV light for 2 min, and the EPR signal was recorded after 3 min. To evaluate the hydroxyl radical scavenging activity of Chaga extracts, the total reaction volume was kept constant, and 1 μL of distilled water was replaced by 1 μL of the extract, resulting in a reaction mixture containing 1 μL DEPMPO, 26 μL distilled water, 1 μL extract, and 2 μL H_2_O_2_. The same protocol was applied to assess the antioxidant activity of liposome-encapsulated extracts, with 1 μL of liposome suspension added instead of the extract. Extracts and liposome suspension were diluted fivefold.

### 3.9. Statistical Analysis

Pearson correlation analysis was performed to evaluate relationships between compound classes and antioxidant activities (DPPH and ^•^OH). Statistical significance of the correlations was assessed using the t-test, and *p*-values < 0.05 were considered significant. This analysis allowed us to determine which compound classes were most strongly associated with radical scavenging efficiency. To further explore the patterns in the dataset and reduce dimensionality, Principal Component Analysis (PCA) was applied. PCA transforms the original set of correlated variables into a smaller number of uncorrelated principal components, enabling visualization of the main sources of variance among Chaga extracts. In this study, all variables were standardized prior to PCA to eliminate the influence of differences in measurement scales and ensure comparability across parameters. PCA was performed using Octave software v.10.3.0, and the results were visualized as biplots to facilitate interpretation of correlations between chemical composition and antioxidant activities.

To differentiate between samples, HCA plots were constructed in Morpheus software [73], based on the Spearman method of cluster agglomeration, adopting the average linkage method. Due to the absence of authentic analytical standards, this approach provides semi-quantitative, signal-based information only and does not represent absolute or relative concentrations of individual compounds.

All experiments were performed in triplicate, and the values presented here are the means of the three independent measurements. The results are expressed as means with standard error (±SE).

## 4. Conclusions

This study systematically evaluated green extraction methods for *Inonotus obliquus* (Chaga) and demonstrated clear links between extraction parameters, chemical composition, and antioxidant activity. UAE proved most effective for recovering phenolic- and triterpenoid-rich extracts, while ScCO_2_-PLE showed selective hydroxyl radical scavenging under sustainable conditions. Among all extracts, UAE-derived samples with 50% and 70% ethanol (Chaga 4 and Chaga 6) exhibited the highest and most balanced antioxidant potential. Incorporation into liposomal formulations effectively preserved DPPH and ^•^OH scavenging activity, particularly for hydroethanolic extracts, highlighting the ability of liposomes to protect bioactive compounds and maintain functional activity. Combining optimized green extraction with liposomal delivery represents a promising strategy for enhancing the stability and antioxidant benefits of Chaga in nutraceutical or therapeutic applications. While these results are encouraging, this study is limited by the focus on in vitro antioxidant activity and the evaluation of only selected extraction methods and solvent ratios. Future research will include optimization of liposome formulations and extraction approaches, followed by biological evaluation in cell culture models and in vivo systems to ensure physiological relevance. These approaches aim to further optimize extract bioactivity and expand the understanding of the potential therapeutic applications of Chaga.

## Figures and Tables

**Table 1:** Characterization of Bioactive Compounds Identified in Nine Chaga Extracts by HPLC-Orbitrap Exploris 120.

No	*t*_R_, min	Molecular Formula, [M–H]^−^	Calculated Mass, *m/z*	Exact Mass, *m/z*	Δ ppm	MS^2^ Fragments, (% Base Peak)	Compound Name	Extract
**1**	0.53	C_6_H_13_O_6_^−^	181.07176	181.07244	−3.74	59.01408(75), 71.01416(59), 89.02478(100), 101.02483(79)	Mannitol	all
**2**	0.55	C_6_H_11_O_7_^−^	195.05103	195.05185	−4.20	59.01411(22), 75.00911(100), 87.00919(18), 99.00924(29), 129.01994(50), 195.05188(68)	Gluconic acid	all
**3**	0.57	C_4_H_5_O_6_^−^	149.00920	149.00985	−4.38	59.01409(53), 72.99344(46), 75.00909(12), 87.00916(90), 103.00414(20), 149.00986(100)	Dihydroxybutanedioic acid	all
**4**	0.61	C_6_H_7_O_7_^−^	191.01973	191.02052	−4.17	85.02984(33), 87.0091(47), 111.00922(100), 129.01984(8)	Isocitric acid	all
**5**	0.61	C_7_H_7_O_8_^−^	219.01464	219.01565	−4.61	69.03485(99), 83.05054(51), 85.02982(76), 99.00910(67), 113.02486(100), 131.03543(51)	2-Hydroxy-4-oxo-1,2,4-butanetricarboxylic acid	all
**6**	0.68	C_8_H_5_O_6_^−^	197.00916	197.00997	−4.11	109.02995(100), 125.02489(5), 153.01997(97), 197.01007(12)	2,5-Dihydroxyterephthalic acid	all
**7**	0.72	C_12_H_15_O_7_^−^	271.08233	271.08339	−3.90	109.02996(100), 121.06644(38), 139.04074(62), 139.0771(34), 227.05774(35), 227.09396(31)	Arbutin	Chaga 2, 5, 7–9
**8**	0.75	C_7_H_5_O_4_^−^	153.01933	153.02002	−4.49	109.03004(100), 153.02011(31)	3,4-Dihydroxybenzoic acid	all
**9**	0.78	C_9_H_5_O_6_^−^	209.00916	209.01014	−4.69	93.03501(7), 121.03004(100), 137.02505(8), 165.02008(45), 209.01004(5)	Benzenetricarboxylic acid	Chaga 1–5, 7–9
**10**	1.01	C_6_H_3_O_5_^−^	154.99860	154.99932	−4.61	67.01929(100), 83.01434(6), 111.00941(64), 111.04633(3), 154.99959(13)	Furandicarboxylic acid	all
**11**	1.04	C_7_H_3_O_7_^−^	198.98840	198.98928	−4.44	67.01925(100), 111.00932(96), 154.99939(17)	Meconic acid	Chaga 1–4, 6–9
**12**	1.46	C_7_H_5_O_3_^−^	137.02442	137.02493	−3.70	137.02499(100)	3,4-Dihydroxybenzaldehyde	all
**13**	2.62	C_9_H_7_O_4_^−^	179.03498	179.03572	−4.12	135.04579(100), 179.03587(26)	Caffeic acid	Chaga 1, 2, 6, 8
**14**	5.67	C_8_H_5_O_5_^−^	181.01425	181.01505	−4.46	93.03501(100), 137.02507(18), 181.01511(37)	4-Hydroxyisophthalic acid	all
**15**	5.76	C_12_H_15_O_7_^−^	271.08233	271.08345	−4.14	138.03302(18), 153.05652(100), 167.03587(10), 196.03871(12), 197.04655(9), 212.07013(22)	2-Hydroxy-1-(hydroxymethyl)ethyl 4-hydroxy-3,5-dimethoxybenzoate	all
**16**	5.82	C_9_H_9_O_5_^−^	197.04555	197.04638	−4.20	123.00948(9), 138.03305(14), 153.05661(5), 166.99947(26), 182.02299(100), 197.04655(12)	Syringic acid	all
**17**	5.99	C_14_H_9_O_6_^−^	273.04046	273.04165	−4.35	167.05093(18), 185.06160(30), 229.05151(33), 273.04156(100)	6,6′-Dihydroxy[1,1′-biphenyl]-3,3′-dicarboxylic acid	all
**18**	6.16	C_12_H_5_O_6_^−^	245.00916	245.01021	−4.28	133.03009(61), 157.03021(100), 189.02017(50), 201.02036(23), 217.01501(24), 245.01038(83)	Gomphilactone	Chaga 3–6, 9
**19**	6.17	C_13_H_5_O_8_^−^	288.99899	289.00011	−3.86	189.02022(16), 201.02042(10), 217.01517(100), 245.01030(41), 289.00015(71)	Phelligridin J	Chaga 3–6, 8, 9
**20**	6.28	C_9_H_5_O_7_^−^	225.00408	225.00509	−4.50	93.03491(100), 137.02498(14), 181.01485(8), 225.00493(56)	Hydroxy-benzenetricarboxylic acid	Chaga 1–4, 6–8
**21**	6.28	C_9_H_5_O_3_^−^	161.02442	161.02512	−4.39	117.03510(100), 161.02510(23)	Umbelliferone	all
**22**	6.29	C_11_H_5_O_7_^−^	249.00408	249.00509	−4.08	117.03514(28), 161.02512(100)	3-Hydroxy-2-oxo-2H-1-benzopyran-4,6-dicarboxylic acid	all
**23**	6.35	C_10_H_9_O_3_^−^	177.05570	177.05637	−3.81	135.04570(4), 177.05637(100)	(E)-4-(3,4-Dihydroxyphenyl)but-3-en-2-one	all
**24**	6.44	C_14_H_15_O_7_^−^	295.08233	295.08354	−4.10	119.05084(65), 163.04097(77), 262.04962(92), 265.03656(20), 277.07303(100), 280.06021(73)	Aquilarin A	Chaga 1–6, 8,9
**25**	6.45	C_23_H_27_O_13_^−^	511.14572	511.14769	−3.86	180.04359(12), 182.02277(18), 195.06709(96), 197.04636(100), 210.05417(16), 225.07782(55)	Dimethoxyhydroquinone syringoyl-hexsode	Chaga 2–9
**26**	6.57	C_12_H_9_O_8_^−^	281.03029	281.03145	−4.13	137.02486(20), 149.06129(79), 193.01796(13), 193.05049(12), 237.04102(19), 281.03119(100)	Benzenetetracarboxylic acid dimethyl ester	Chaga 1–7, 9
**27**	6.60	C_10_H_7_O_4_^−^	191.03498	191.03580	−4.30	132.02229(33), 147.04602(10), 176.01225(100), 191.03641(27)	Scopoletin	all
**28**	6.65	C_12_H_7_O_8_^−^	279.01464	279.01580	−4.17	132.02231(6), 147.04581(12), 176.01222(40), 191.03578(100)	Spinochrome A	all
**29**	6.72	C_16_H_15_O_9_^−^	351.07216	351.07336	−3.42	135.04578(16), 179.03586(11), 182.02275(9), 197.04654(11), 223.02573(100)	Auxarthrol C	all
**30**	6.73	C_13_H_9_O_5_^−^	245.04555	245.04659	−4.24	135.04564(5), 159.04578(100), 201.05643(45)	Hispidin	Chaga 1–6, 8,9
**31**	6.79	C_12_H_9_O_4_^−^	217.05063	217.05160	−4.44	133.03008(61), 155.10849(11), 171.10339(10), 175.04076(16), 217.05165(100)	Inotilone	Chaga 1–6, 8,9
**32**	6.88	C_15_H_9_O_7_^−^	301.03538	301.03673	−4.48	167.05087(7), 195.04597(26), 213.05667(53), 239.03612(15), 257.04657(100), 301.03659(63)	4-(2,6-Dihydroxybenzoyl)-3-formyl-5-hydroxybenzoic acid	Chaga 1–8
**33**	6.99	C_9_H_15_O_4_^−^	187.09758	187.09840	−4.39	125.09777(100)	Azelaic acid	all
**34**	7.80	C_12_H_11_O_4_^−^	219.06628	219.06716	−3.99	135.04575(100), 161.02509(5), 177.05649(3)	Hispolon	Chaga 2–6, 9
**35**	7.81	C_8_H_7_O_2_^−^	135.04515	135.04577	−4.55	NA	4-Methoxybenzaldehyde	Chaga 3–6, 9
**36**	7.82	C_20_H_11_O_8_^−^	379.04594	379.04740	−3.84	229.01538(18), 269.01025(100), 307.06244(17), 335.05743(35), 351.05225(14)	Phelligridin D	all
**37**	9.04	C_17_H_25_O_4_^−^	293.17583	293.17724	−4.81	117.03513(24), 161.02515(34), 205.01514(42), 220.14780(42), 221.15555(100), 236.10634(86)	Gingerol	all
**38**	10.32	C_18_H_31_O_3_^−^	295.22787	295.22919	−4.48	119.05076(22), 163.04105(15), 171.10347(48), 277.21844(100)	(12Z)-10-Oxo-12-octadecenoic acid	all
**39**	10.56	C_30_H_49_O_4_^−^	473.36363	473.36524	−3.40	473.36551(100)	Inonoterpene A	Chaga 1–6, 8,9
**40**	10.65	C_30_H_47_O_5_^−^	487.34290	487.34473	−3.77	209.15549(11), 425.34958(4), 443.35507(17), 469.33310(5), 487.34460(100)	Maslinic acid	all
**41**	10.67	C_31_H_49_O_4_^−^	485.36363	485.36535	−3.53	485.36624(100)	Hispindic acid B	all
**42**	11.02	C_31_H_47_O_4_^−^	483.34798	483.35008	−4.34	357.28162(4), 367.26627(6), 369.28171(8), 439.37103(8), 483.35114(100)	Hispindic acid A	Chaga 3–6, 8, 9
**43**	11.91	C_39_H_55_O_5_^−^	603.40550	603.40758	−3.44	161.02512(3), 603.40723(100)	Betulin-3-caffeate	all
**44**	11.94	C_30_H_45_O_3_^−^	453.33742	453.33902	−3.52	453.33911(100)	Pinicolic acid	all
**45**	11.94	C_28_H_43_O_6_^−^	475.30651	475.30811	−3.37	315.27060(23), 369.31760(15), 413.30756(31), 431.31805(100)	Polyporusterone B	all
**46**	12.10	C_30_H_47_O_3_^−^	455.35307	455.35469	−3.55	455.35474(100)	Betulinic acid	all

NA—not available.

**Table 2:** Antioxidant Activities of Chaga extracts against DPPH and ^•^OH radical. Extracts were obtained using three extraction methods: maceration, ultrasound-assisted extraction (UAE), and supercritical CO_2_ extraction with pressured liquid extraction (ScCO_2_-PLE), and three solvent systems: distilled water, 50% ethanol, and 70% ethanol.

Extract	Extraction Method	Solvent	DPPH Scavenging (%)	OH Scavenging (%)
Chaga 1	Maceration	Water	56.2 ± 0.1	31.5 ± 1.2
Chaga 2	UAE	Water	65.3 ± 0.1	45.3 ± 1.2
Chaga 3	Maceration	50% Ethanol	98.8 ± 0.3	90.6 ± 1.0
Chaga 4	UAE	50% Ethanol	98.4 ± 0.3	92.7 ± 1.0
Chaga 5	Maceration	70% Ethanol	94.3 ± 0.3	91.6 ± 1.0
Chaga 6	UAE	70% Ethanol	98.5 ± 0.3	91.7 ± 1.0
Chaga 7	ScCO_2_-PLE	Water	38.1 ± 0.1	69.7 ± 1.2
Chaga 8	ScCO_2_-PLE	50% Ethanol	91.8 ± 0.3	89.6 ± 1.0
Chaga 9	ScCO_2_-PLE	70% Ethanol	15.7 ± 0.3	89.5 ± 1.0

**Table 3:** Correlation coefficients (r) between selected individual metabolites and antioxidant activities toward DPPH and hydroxyl (^•^OH) radicals in Chaga extracts. Pearson correlation coefficients for key metabolites (whose structural formulas are given in Figure S3) highlight the contribution of specific polyphenolic, terpenoid, and phenolic acid compounds to radical scavenging activity. Significant correlations are indicated as *p* < 0.05 (*), *p* < 0.01 (**), and *p* < 0.001 (***). Strongly positive correlations identify compounds that are likely major contributors to antioxidant potential, while strongly negative correlations indicate metabolites that may suppress or counteract antioxidant efficiency.

	r (DPPH)	r (^•^OH)
Azelaic acid	−0.94 ***	−0.86 **
Dihydroxybutanedioic acid	−0.82 **	0.81 **
Hispolon	0.73 *	0.81 **
4-Methoxybenzaldehyde	0.72 *	0.80 *
Hispidin	0.66	0.79 *
Polyporusterone B	0.63	−0.79 *
4-Hydroxyisophthalic acid	−0.62	−0.79 *
Hispindic acid B	0.59	0.78 *
Gluconic acid	−0.58	−0.77 *
Gingerol	−0.58	0.75 *

**Table 4:** Antioxidant Activities of liposomes with Chaga extracts against DPPH and ^•^OH radical. Extracts (Chaga 4, 6, and 7) were obtained using two extraction methods: ultrasound-assisted extraction (UAE) and supercritical CO_2_ extraction with pressurized liquid extraction (ScCO_2_-PLE), and three solvent systems: distilled water, 50% ethanol, and 70% ethanol.

Sample	Extraction Method	Solvent	DPPH Scavenging (%)	^•^OH Scavenging (%)
Liposomes + Chaga 7	ScCO_2_-PLE	Water	2.3 ± 0.7	16.1 ± 2.1
Liposomes + Chaga 4	UAE	50% Ethanol	93.5 ± 0.5	82.9 ± 1.6
Liposomes + Chaga 6	UAE	70% Ethanol	87.6 ± 0.5	86.1 ± 1.6

**Table 5:** Comparative Antioxidant Activities of Chaga Extracts and Chaga-Loaded Liposomes Against DPPH and ^•^OH Radicals.

Sample	Extraction Method	Solvent	DPPH Scavenging (%)	^•^OH Scavenging (%)
Chaga 7	ScCO_2_-PLE	Water	38.1 ± 0.1	69.7 ± 1.2
Liposomes + Chaga 7	ScCO_2_-PLE	Water	2.3 ± 0.7	16.1 ± 2.1
Chaga 4	UAE	50% Ethanol	98.4 ± 0.3	92.7 ± 1.0
Liposomes + Chaga 4	UAE	50% Ethanol	93.5 ± 0.5	82.9 ± 1.6
Chaga 6	UAE	70% Ethanol	98.5 ± 0.3	91.7 ± 1.0
Liposomes + Chaga 6	UAE	70% Ethanol	87.6 ± 0.5	86.1 ± 1.6

**Table 6:** Overview of Chaga extracts, indicating the extraction method applied and the solvent used for each labeled sample.

Extract	Extraction Method	Solvent
Chaga 1	Maceration	Water
Chaga 2	UAE	Water
Chaga 3	Maceration	50% Ethanol
Chaga 4	UAE	50% Ethanol
Chaga 5	Maceration	70% Ethanol
Chaga 6	UAE	70% Ethanol
Chaga 7	ScCO_2_-PLE	Water
Chaga 8	ScCO_2_-PLE	50% Ethanol
Chaga 9	ScCO_2_-PLE	70% Ethanol

## Data Availability

The original contributions presented in this study are included in this article/Supplementary Materials; further inquiries can be directed to the corresponding authors.

## Supplementary Materials

The following supporting information can be downloaded at: https://www.mdpi.com/article/10.3390/molecules31010146/s1, Figure S1: Schematic overview of the experimental workflow; Figure S2: Representative base peak chromatogram of one of the Chaga extracts (Chaga 6, 70% Ethanol, UAE); Table S1: Peak areas of identified compounds in nine Chaga extracts determined by HPLC–LTQ Orbitrap MS; Figure S3: Chemical structures of the key metabolites highlighting the contribution of specific bioactive compounds to radical scavenging activity; Figure S4: Representative EPR spectra illustrating the decrease of: (A) DPPH signal in water; (B) DPPH signal in 70% Ethanol; (C) DEPMPO-OH adduct signal, upon addition of extracts obtained by different extraction methods (UAE, ScCO_2_-PLE, and maceration), relative to the control; Figure S5: Line plot of Pearson correlation coefficients (r) between antioxidant activities (DPPH and ^•^OH) and PCA components (PC1–PC8); Figure S6: Heatmap of correlations between DPPH and ^•^OH activities and the first five PCA components (PC1–PC5); Figure S7: PCA score plot of Chaga extracts (PC1 vs. PC2) colored by DPPH activity; Figure S8: PCA score plot of Chaga extracts (PC1 vs. PC2) colored by ^•^OH activity; Table S2: Encapsulation efficiency and retention of liposomes with Chaga extracts over time; Table S3: Size and zeta potential of liposomes with Chaga extracts over time.

## Abbreviations

The following abbreviations are used in this manuscript:

UAE	Ultrasound-Assisted Extraction
ScCO_2_-PLE	Supercritical CO_2_-Pressurized Liquid Extraction
HPLC	High-Performance Liquid Chromatography
LC	Liquid Chromatography
MS	Mass Spectrometry
PCA	Principal Component Analysis
EPR	Electron Paramagnetic Resonance
*AA*	Antioxidant Activity
DPPH	2,2-diphenyl-1-picrylhydrazyl
DEPMPO	5-(Diethoxyphosphoryl)-5-methyl-1-pyrroline N-oxide
^•^OH	Hydroxyl radical
TEM	Transmission Electron Microscopy
DLS	Dynamic Light Scattering
ELS	Electrophoretic Light Scattering
SE	Standard Error
