# Supplementary material for: Green Extraction and Liposomal Encapsulation of Inonotus obliquus (Chaga) Extracts: Comparative Phytochemical and Antioxidant Analysis
Source: Molecules. 2026 Jan 1;31(1):146. doi: 10.3390/molecules31010146 (PMC12788109; doi:10.3390/molecules31010146)
Supplement: Supplementary file 1 [file molecules-31-00146-s001.zip › molecules-4037191-supplementary.pdf]

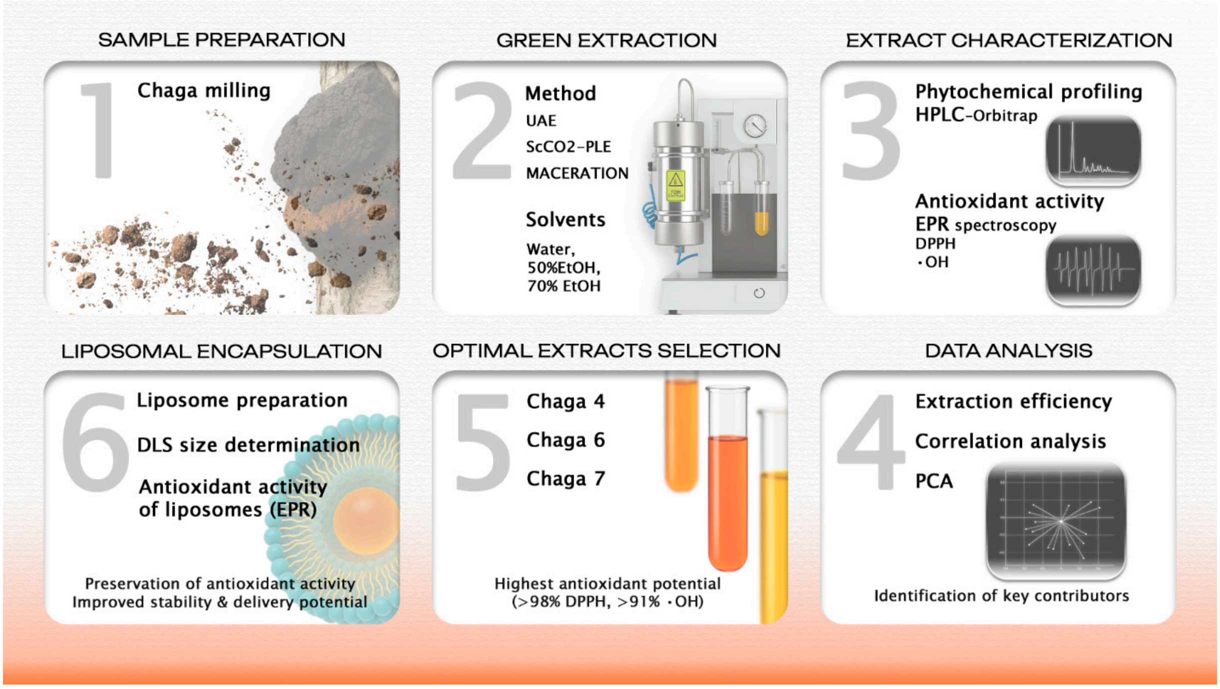

Figure S1. Schematic overview of the experimental workflow

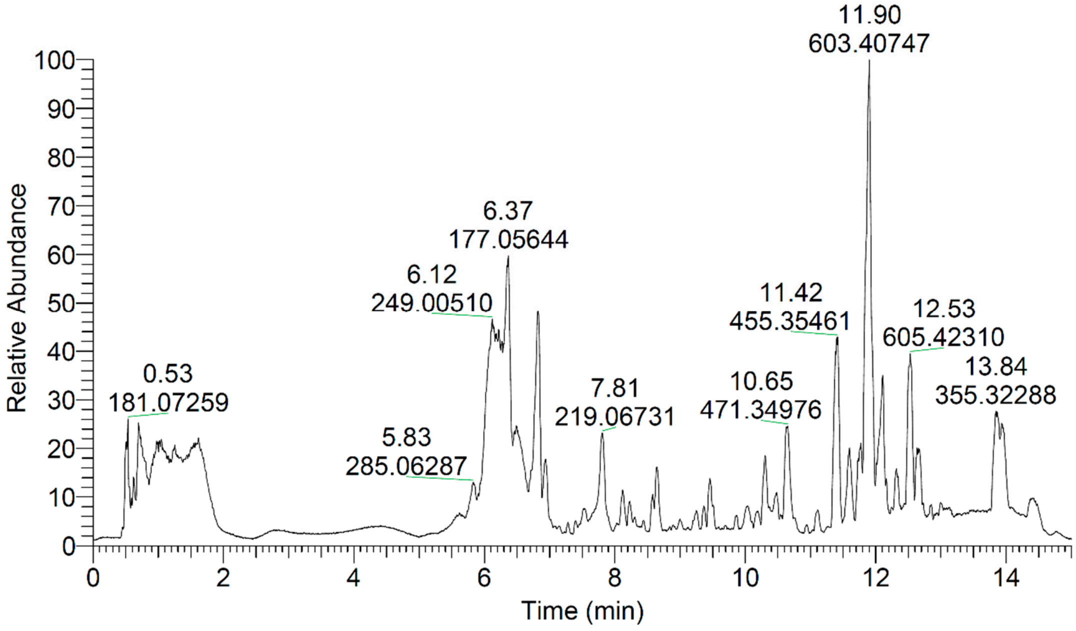

Figure S2. Representative base peak chromatogram of one of the Chaga extracts (Chaga 6, 70% Ethanol, UAE).

Table S1. Peak areas of identified compounds in nine Chaga extracts determined by HPLC–LTQ Orbitrap MS.

Compound name	Chaga 1	Chaga 2	Chaga 3	Chaga 4	Chaga 5	Chaga 6	Chaga 7	Chaga 8	Chaga 9
**Mannitol**	272672566	173657133	162535365	128208761	134305816	182026315	164939230	145844013	76015966
**Gluconic acid**	32299678	31597915	22905408	23484399	19987234	27053776	99471344	26362061	17566160
**Dihydroxybutanedioic acid**	4153734	6118882	4911946	6750889	6462742	7199494	64864150	40570165	74260802
**Isocitric acid**	98667314	86035310	96335677	98142482	93449046	119626178	57515954	62522243	70852686
**2-Hydroxy-4-oxo-1,2,4-butanetricarboxylic acid**	100717098	91593341	85214198	84530675	108792760	97469917	58324848	59192223	57136097
**2,5-Dihydroxyterephthalic acid**	697419856	598701040	655403181	502776960	418173273	808849082	43905146	433744932	242383535
**Arbutin**		2593792			8551716		4626545	6230475	3424245
**3,4-Dihydroxybenzoic acid**	146352592	266827053	99588749	223549361	78968841	120501428	4261392	83061959	69793260
**Benzenetricarboxylic acid**	142317954	145776201	92681788	82773452	3217632		20022956	2091642	1442734
**Furandicarboxylic acid**	242831736	189448653	157496369	190245060	20983205	114052914	54858691	170347450	26581605
**Meconic acid**	513182993	432324162	470463506	431921259		716924181	278503773	343147904	345104
**3,4-Dihydroxybenzaldehyde**	186156026	207749149	541081599	380587756	430420781	614432159	307512616	464413680	299604496
**Caffeic acid**	22335278	43655205				137820543		60919104	
**4-Hydroxyisophthalic acid**	127058798	89563558	85803204	37547971	88942416	308313165	398161616	31268874	93359903
**2-Hydroxy-1-(hydroxymethyl)ethyl 4-hydroxy-3,5-dimethoxybenzoate**	95226862	88037441	77607618	79205456	60726105	194624318	93643660	82091187	32941135
**Syringic acid**	349563942	275575484	255612122	284395412	173628863	377526365	439897908	254149982	29478352
**6,6′-Dihydroxy[1,1′-biphenyl]-3,3′-dicarboxylic acid**	136927576	93559206	59841138	155795931	45036161	146662220	49808363	85931626	41750446
**Gomphilactone**			322921129	195465717	87096303	191781363			71936530
**Phelligridin J**			7973493	3707786	150789509	793273611		377789286	115142145
**Hydroxy-benzenetricarboxylic acid**	78837018	160148353	431084146	121937125		499863567	667219555	77995663	
**Umbelliferone**	606599412	551027417	609113572	595971488	130203690	993973066	582023873	508937776	147153997
**3-Hydroxy-2-oxo-2H-1-benzopyran-4,6-dicarboxylic acid**	1190324710	1093262825	1281451186	1353129173	249534918	2411005696	1260255052	1127990958	263822096
**(E)-4-(3,4-Dihydroxyphenyl)but-3-en-2-one**	99407493	121051884	954349714	713064484	413563258	1225623269	24225796	644483708	632870257
**Aquilarin A**	47627966	52297963	114858190	99130961	62596941	129568928		98700877	68817459
**Dimethoxyhydroquinone syringoyl-hexsode**		295999055	258963443	262536140	131147876	286666089	413257990	279374614	93754734
**Benzenetetracarboxylic acid dimethyl ester**	166119747	123337218	197098078	196425142	15170119	317964460	173102569		12590854
**Scopoletin**	113964571	133139784	154852664	157792227	8852702	248405601	128178393	67504638	21252847
**Spinochrome A**	290961337	262846038	348308669	318143919	27705517	566082944	263637327	291312244	64744400

**Auxarthrol C**	116462390	130306979	148503155	136733582	15069025	241721974	2717662	93586284	24547817
**Hispidin**	15457764	32528620	181059376	223176585	109855411	219225573		122551321	46454086
**Inotilone**	6922214	15682863	68594794	80050513	34883052	101970542		138191803	149979996
**4-(2,6-Dihydroxybenzoyl)-3-formyl-5-hydroxybenzoic acid**	201575893	203116560	237940896	218423013	19829058	334509817	271011639	247306635	
**Azelaic acid**	98133669	66332995	54441293	49431013	27894766	65254197	104437831	94635470	86460112
**Hispolon**		5960059	238581915	216511561	119703193	547274485			4475626
**4-Methoxybenzaldehyde**			113585091	93349330	59294680	185151079			3205980
**Phelligridin D**	6111100	14901658	81378147	65612138	145010146	230443593	24412656	43008869	152628868
**Gingerol**	35619847	27222873	19931962	21176313	17327166	15395067	151334013	42166298	23224004
**(12Z)-10-Oxo-12-octadecenoic acid**	10608286	20421272	217982701	146019215	62701790	248084280	30118513	165218554	113199772
**Inonoterpene A**	377532	1362539	10423283	11820928	2546861	22001874		7429318	4688416
**Maslinic acid**	2580369	7567268	44113876	44087567	37507359	136233658	2327630	37356837	66082279
**Hispindic acid B**	641243	3105152	32485697	33129474	6740110	38858550	302484	25847361	8074129
**Hispindic acid A**			3276327	2720990	778667	5645897		1014550	1666808
**Betulin-3-caffeate**	1772734	4763196	785923268	469375832	396065648	1455739980	181097141	152210710	220792898
**Pinicolic acid**	10191986	36554767	128413282	143281451	63220705	284790213	9495826	312261446	102955951
**Polyporusterone B**	11967672	28849621	314734334	222301786	24337081	492909927	6818291	110177654	41625933
**Betulinic acid**	48909485	110397791	292316173	226071891	211521965	547914840	21473535	323686210	219253748

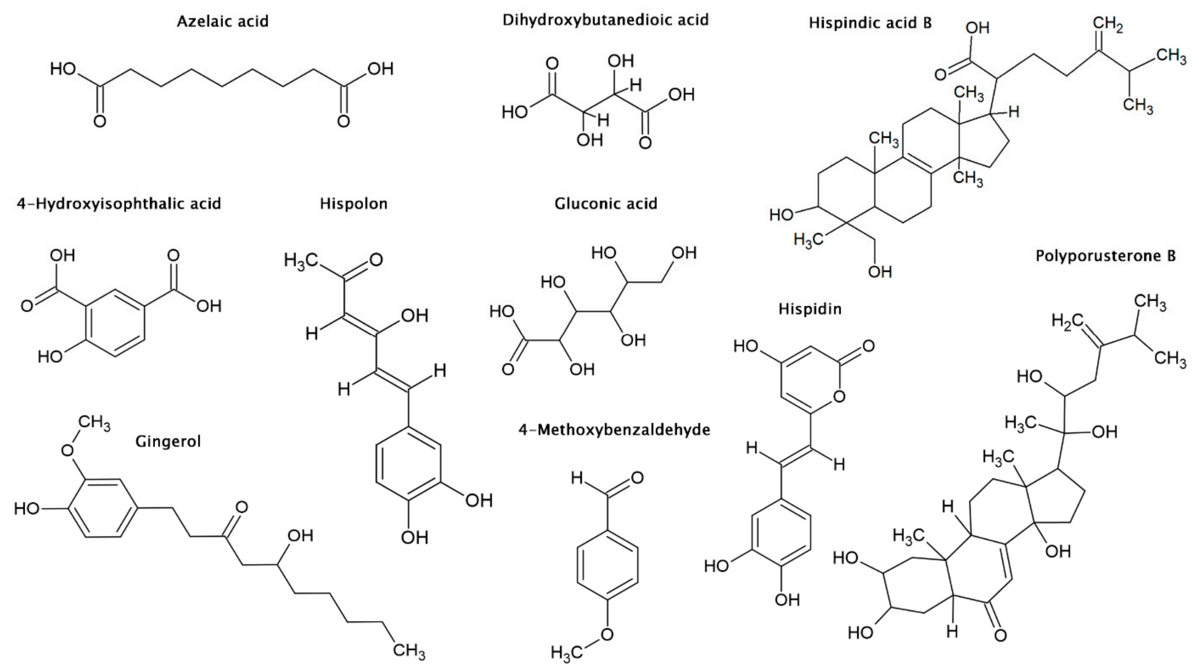

Figure S3. Chemical structures of the key metabolites highlighting the contribution of specific bioactive compounds to radical scavenging activity.

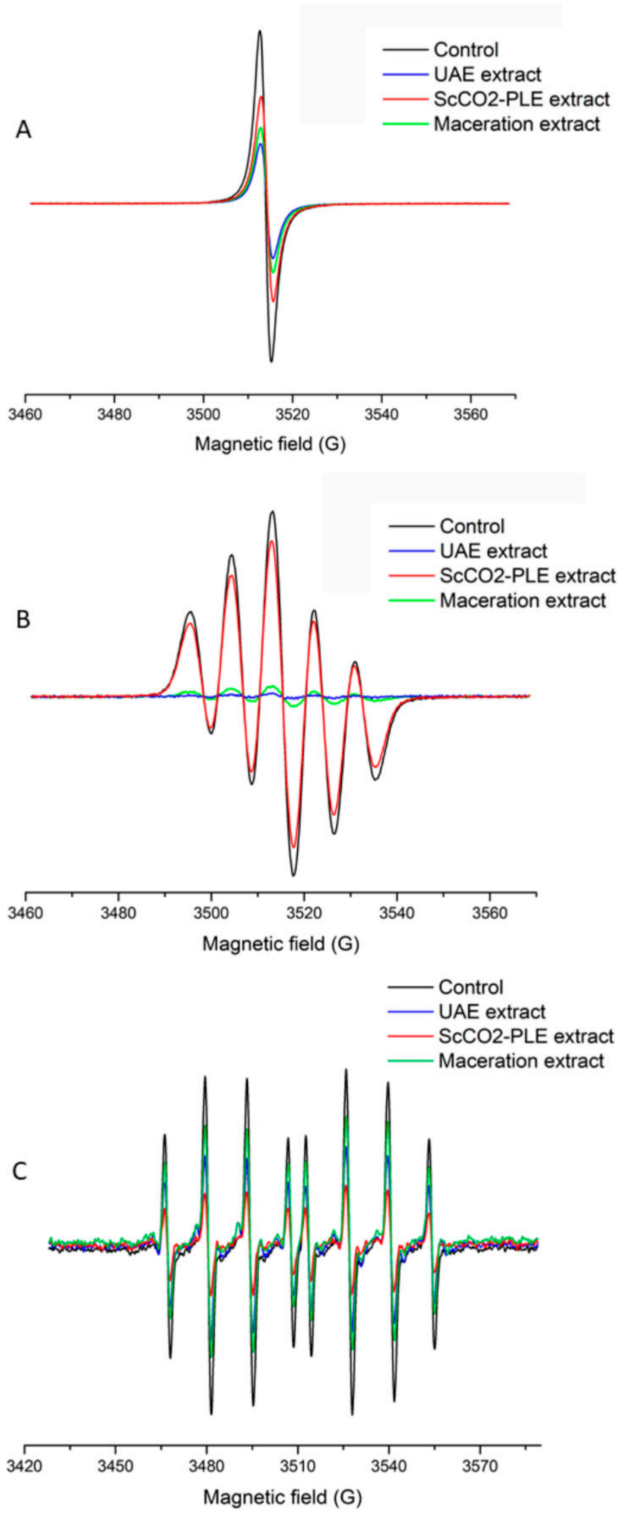

Figure S4. Representative EPR spectra illustrating the decrease of: (A) DPPH signal in water; (B) DPPH signal in 70% Ethanol; (C) DEPMPO-OH adduct signal, upon addition of extracts obtained by different extraction methods (UAE, $ScCO_2$-PLE, and maceration), relative to the control.

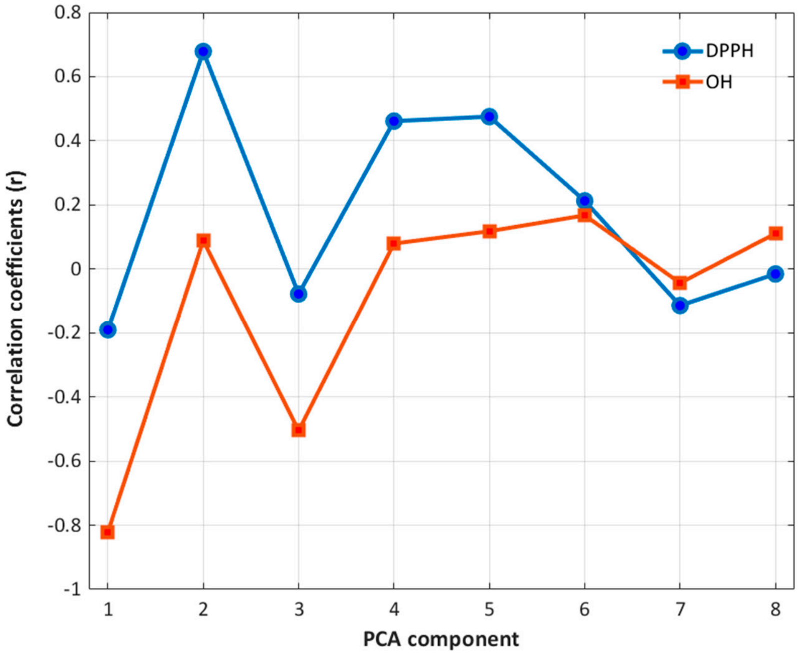

Figure S5. Line plot of Pearson correlation coefficients (r) between antioxidant activities (DPPH and •OH) and PCA components (PC1–PC8). DPPH activity (blue line) shows the strongest positive correlation with PC2 (r ≈ 0.68), while •OH scavenging (orange line) exhibits a pronounced negative correlation with PC1 (r ≈ –0.82). This indicates that the main chemical variance captured by the first two components is directly linked to antioxidant behavior.

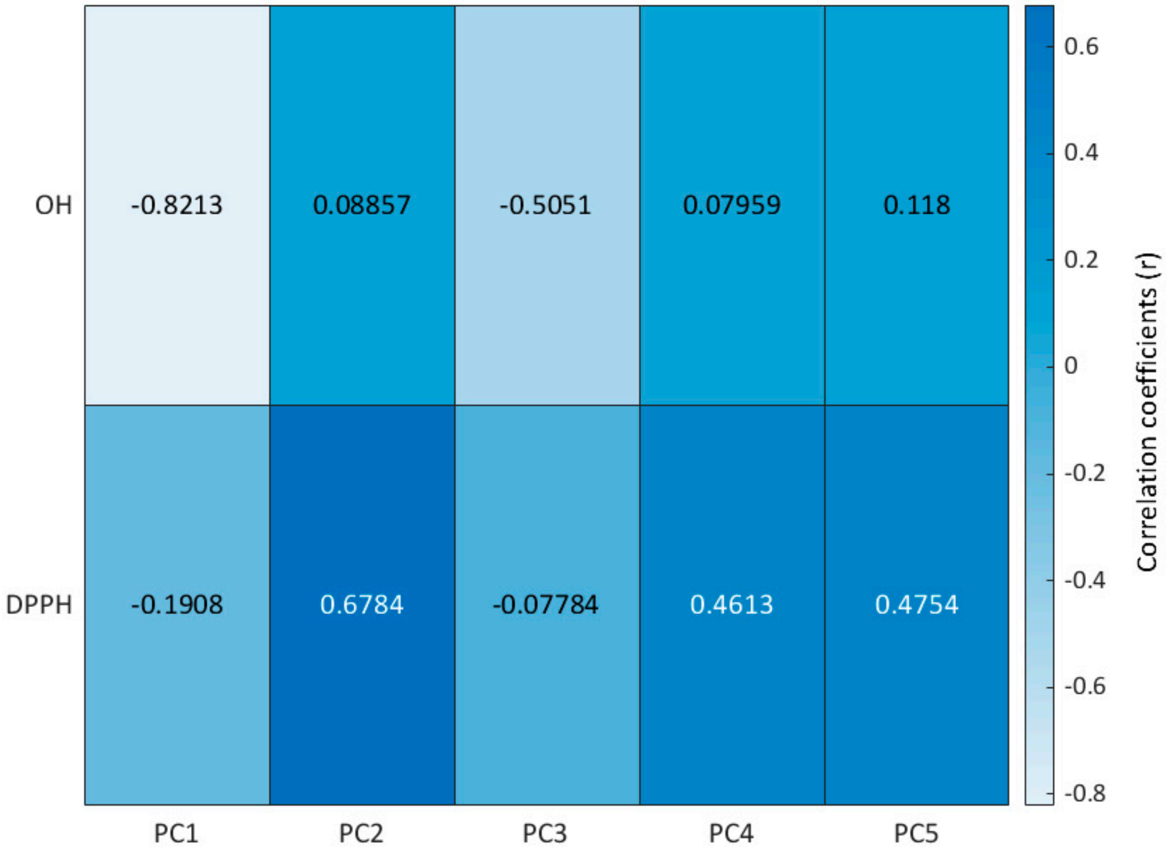

Figure S6. Heatmap of correlations between DPPH and •OH activities and the first five PCA components (PC1–PC5). Darker shades indicate stronger positive correlations, whereas lighter shades indicate stronger negative correlations. DPPH activity shows a strong positive correlation with PC2, while •OH activity is strongly negatively correlated with PC1, in agreement with the line plot trends.

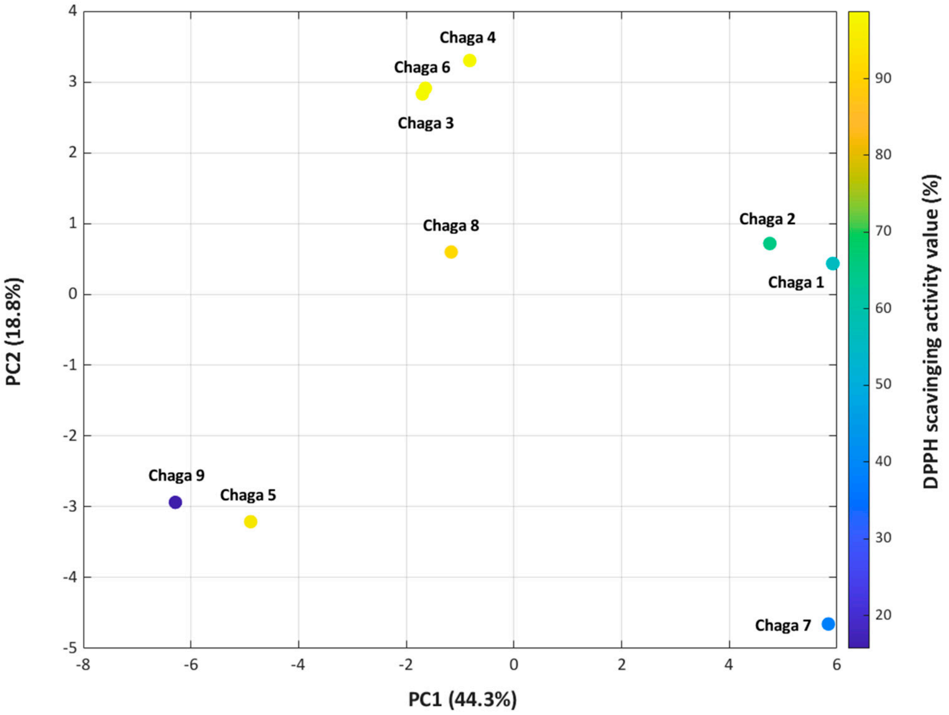

Figure S7. PCA score plot of Chaga extracts (PC1 vs. PC2) colored by DPPH activity. High DPPH scavenging extracts (yellow) cluster at higher PC2 scores, while low-activity extracts (blue) lie at lower PC2 scores, showing PC2 captures DPPH-related chemical variation.

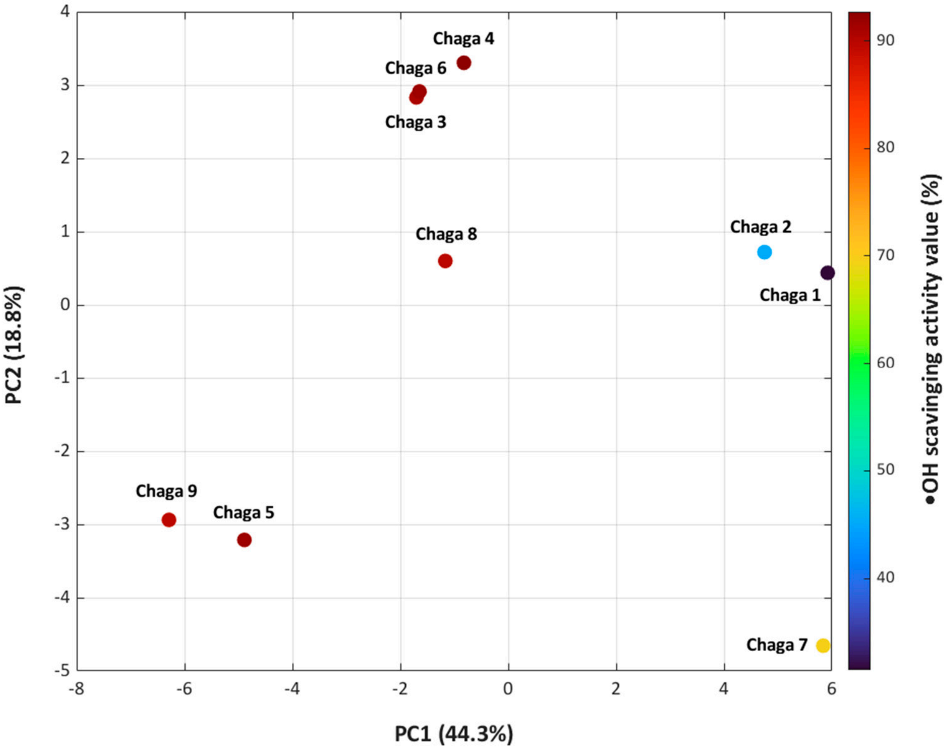

Figure S8. PCA score plot of Chaga extracts (PC1 vs. PC2) colored by •OH activity. High •OH scavenging extracts (red) cluster at negative PC1 scores, and low-activity extracts (blue) cluster at positive PC1 scores, confirming the strong inverse relationship with PC1.

Table S2. Encapsulation efficiency and retention of liposomes with Chaga extracts over time

Liposome formulation	EE (%) Day 0	EE (%) Day 7	Retention (%)
**Liposomes + Chaga 7**	85.31 ± 0.12	82.74 ± 0.19	97.04
**Liposomes + Chaga 4**	86.58 ± 0.29	83.17 ± 0.26	96.09
**Liposomes + Chaga 6**	83.15 ± 0.25	82.11 ± 0.21	98.77

Values are expressed as mean ± SD (n = 3). Retention (%) was calculated according to the equation (1)

Table S3. Size and zeta potential of liposomes with Chaga extracts over time

Liposome formulation	Size (nm) Day 0	Size (nm) Day 7	Δ Size (%)	Zeta potential (mV) Day 0	Zeta potential (mV) Day 7	Δ Zeta (%)
**Liposomes + Chaga 7**	153 ± 1.8	158 ± 2.0	+3.3	−25.0 ± 0.5	−24.8 ± 0.4	0.8
**Liposomes + Chaga 4**	160 ± 2.5	169 ± 3.1	+5.6	−24.1 ± 0.6	−23.7 ± 0.9	1.7
**Liposomes + Chaga 6**	175 ± 2.3	181 ± 2.4	+3.4	−30.7 ± 0.5	−29.5 ± 0.8	3.9

Values are expressed as mean ± SD (n = 3). Δ (%) represents the relative change between Day 0 and Day 7.
